# Supplementary material for: A flexible optoacoustic blood ‘stethoscope’ for noninvasive multiparametric cardiovascular monitoring
Source: Nat Commun. 2023 Aug 4;14:4692. doi: 10.1038/s41467-023-40181-5 (PMC10403590; doi:10.1038/s41467-023-40181-5)
Supplement: Supplementary file 1 — Supplementary Information [file 41467_2023_40181_MOESM1_ESM.docx]

**Supplementary Information for**

**A flexible optoacoustic blood ‘stethoscope’ for non-invasive multiparametric cardiovascular monitoring**

Haoran Jin^1,2,†^, Zesheng Zheng^1,3,†^, Zequn Cui^4^, Ying Jiang^4^, Geng Chen^4^, Wenlong Li^4^, Zhimin Wang^5^, Jilei Wang^4^, Chuanshi Yang^1^, Weitao Song^1^, Xiaodong Chen^4,*^, Yuanjin Zheng^1,*^

^1^ School of Electrical and Electronic Engineering, Nanyang Technological University, Singapore 639​798

^2^ The State Key Laboratory of Fluid Power and Mechatronic Systems, School of Mechanical Engineering, Zhejiang University, Hangzhou, China 310027

^3^ Institute of Microelectronics, Agency for Science, Technology and Research, Singapore 138634

^4^ School of Materials Science and Engineering, Nanyang Technological University, Singapore 639798

^5^ School of Physical and Mathematical Sciences, Nanyang Technological University, Singapore 637371

†These authors contributed equally to this work.

*Correspondence should be addressed to X.C. (email: [chenxd@ntu.edu.sg](mailto:chenxd@ntu.edu.sg)) and Y.Z. (email: [yjzheng@ntu.edu.sg](mailto:yjzheng@ntu.edu.sg)).

**Supplementary Figures**


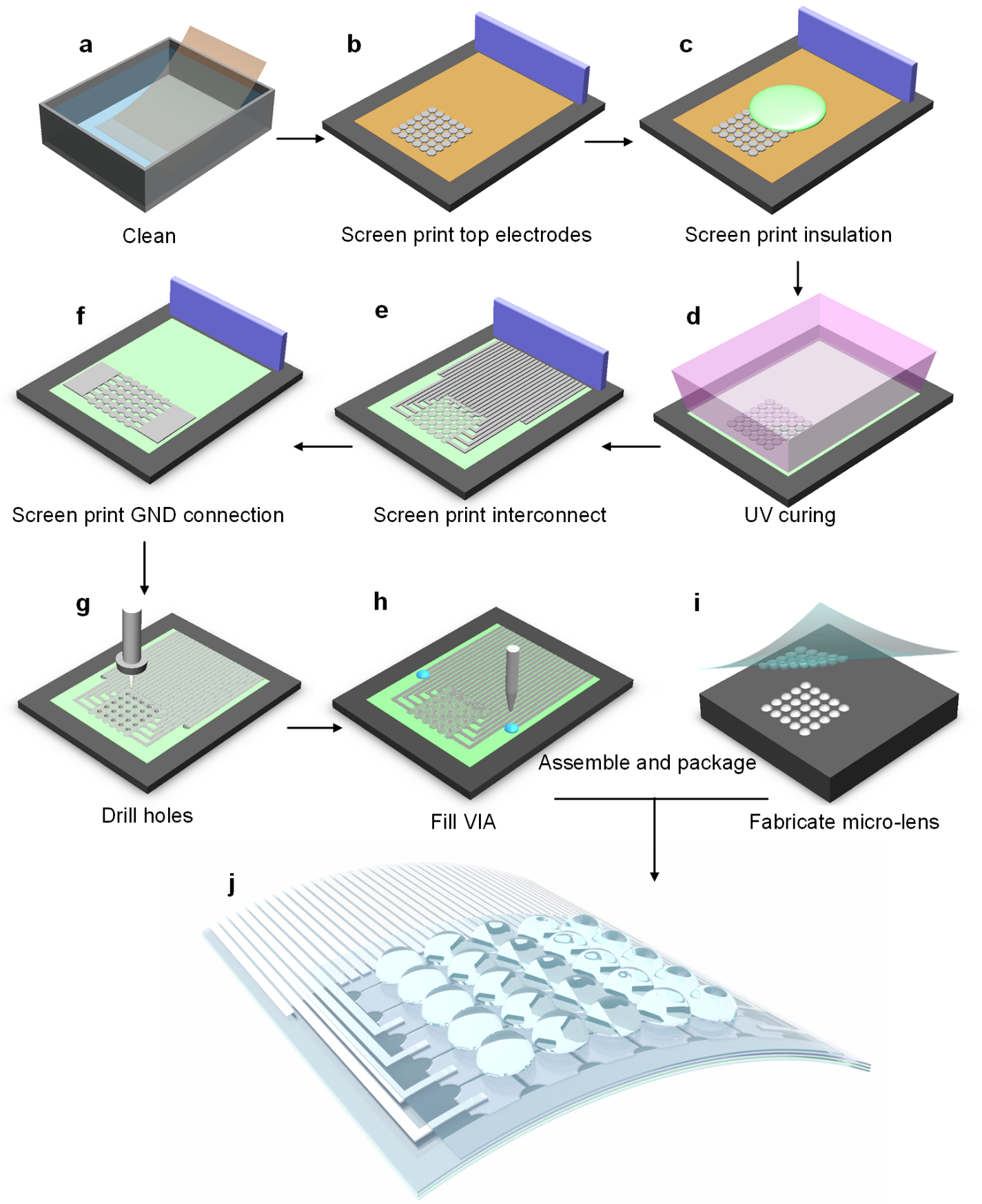


**Supplementary Figure 1. Fabrication of the flexible blood stethoscope. a,** Clean PVDF film. **b,** Screenprint top electrode layer on PVDF film. **c,** Screenprint the insulation layer on PVDF film. **d**, UV cure the insulation layer. **e**, Screenprint interconnection layer on the insulation layer of PVDF film. **f,** Evaporate the bottom ground (GND) electrode layer on the backside of PVDF film. **g,** Drill pinholes with an electronic cutter. **h,** Fill the via hole with conductive silver epoxy. **i,** Fabricate micro-lens with mould. **j**, The micro-lens array is mounted on top of the as-fabricated PVDF acoustic sensor array secured by applying PDMS precursor on the edge of the micro-lens array to achieve the blood stethoscope.

**
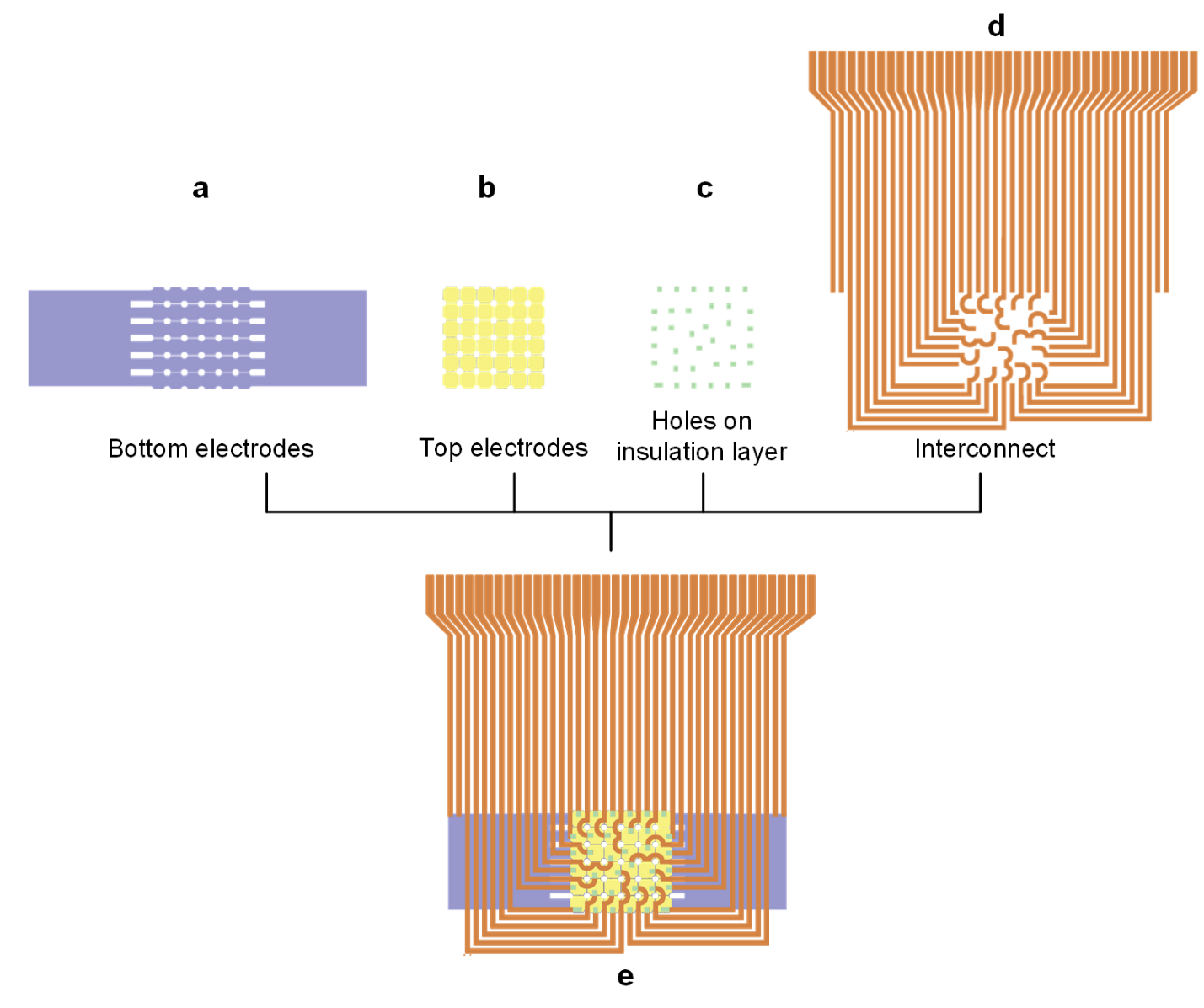
**

**Supplementary Figure 2. Electrodes design. a,** Bottom ground electrodes. **b,** Top electrodes. **c,** Insulation holes. **d,** Interconnection layer. **e,** The integrated multilayer electrodes.

**
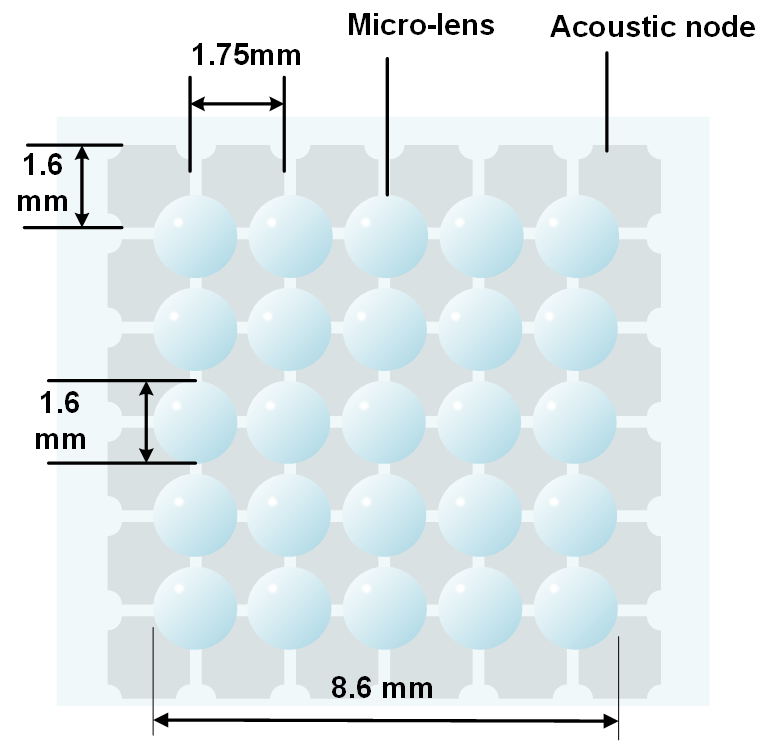
**

**Supplementary Figure 3. The dimensions of microlens and acoustic nodes.**

**
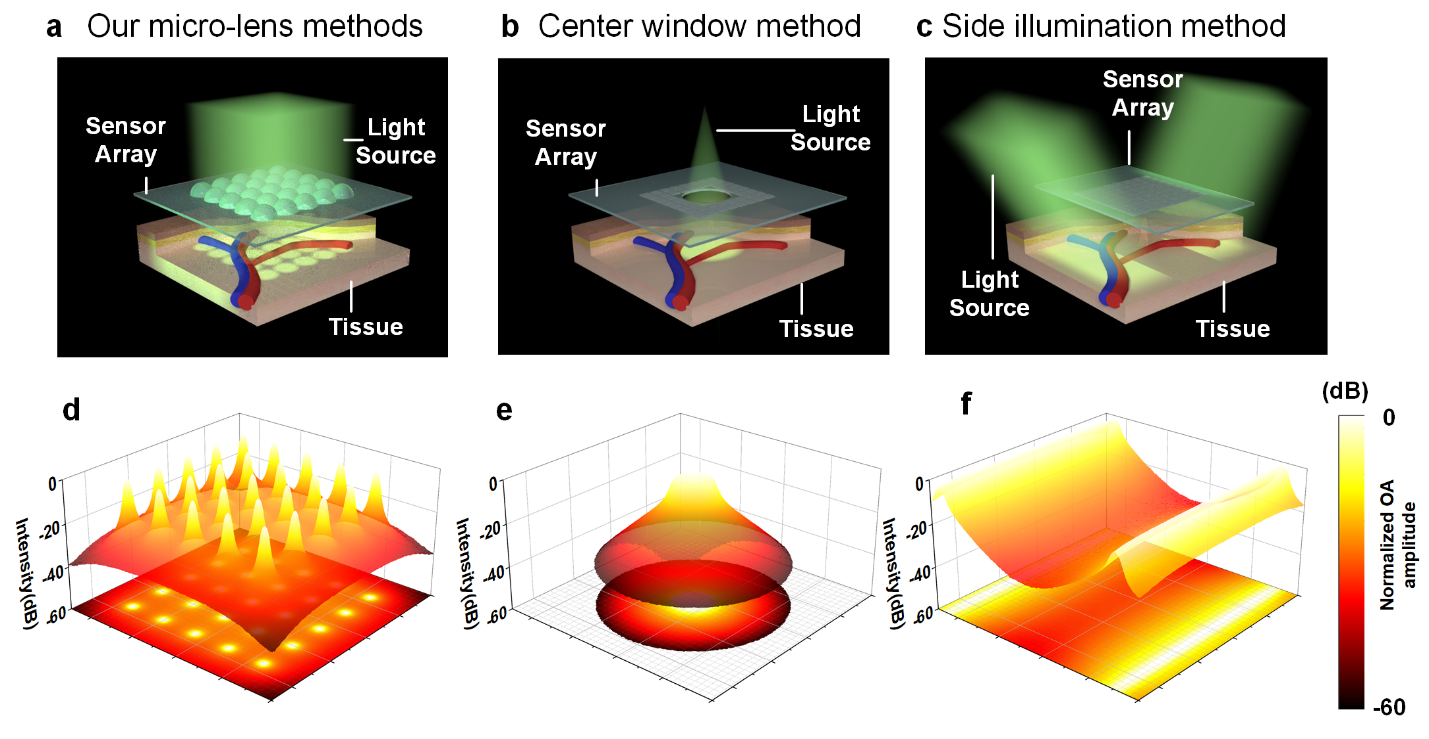
**

**Supplementary Figure 4. Monte Carlo simulation of our micro-lens method with other methods for Optical illumination at wavelength 1000 nm. a-c,** Schematic diagram of different optical element methods. Our micro-lens array method (**a**) is compared with other classical methods including the center window method (**b**) and the side illumination method (**c**). **d-e,** The 1000 nm light fluence distributions after penetrating 1 mm tissue (The light transmission media is assumed to be dermis tissue with optical scattering and absorption coefficients of 97 cm^-1^ and 0.24 cm^-1^, at 1000 nm wavelength, respectively). Our micro-lens array method (**d**) shows a larger effective illumination area (determined by -20 dB illumination intensity) than the center window method (**e**) and the side illumination method (**f**).


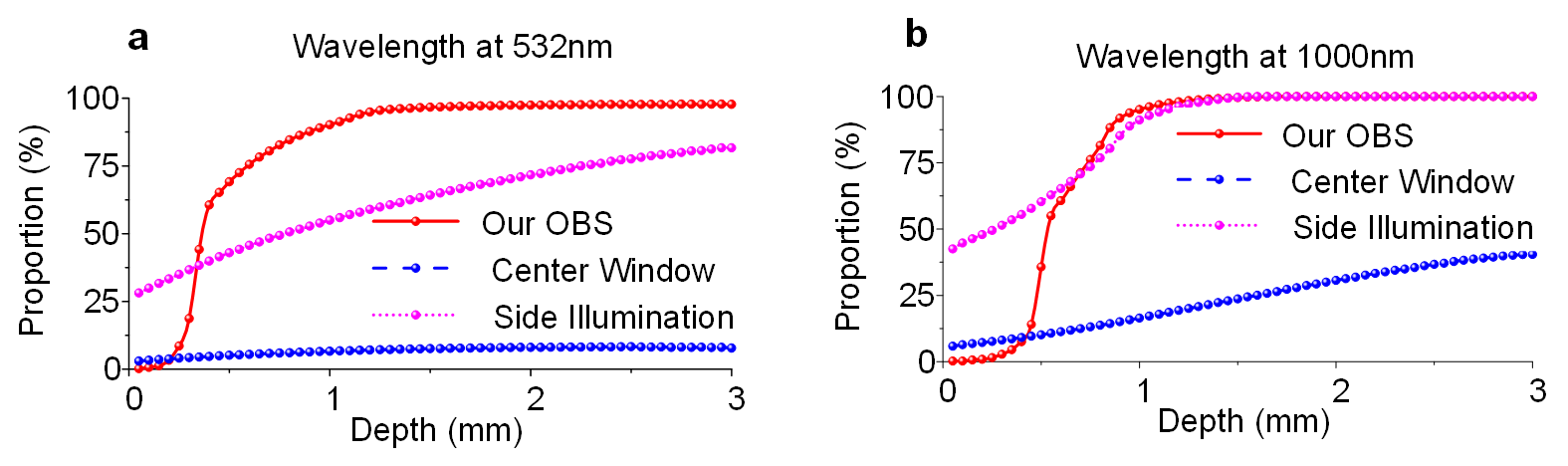


**Supplementary Figure 5.** The proportion of effective illumination area at different tissue depths with wavelengths, 532 nm (**a**) and 1000 nm (**b**).


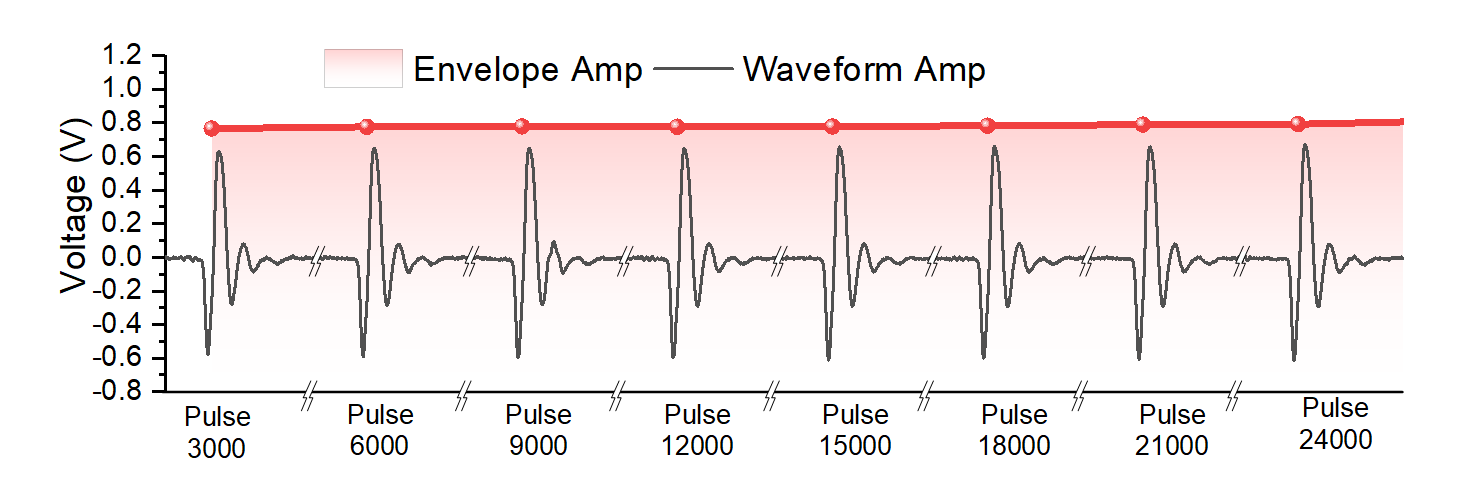


**Supplementary Figure 6.** **The reusability of optoacoustic stethoscope .**


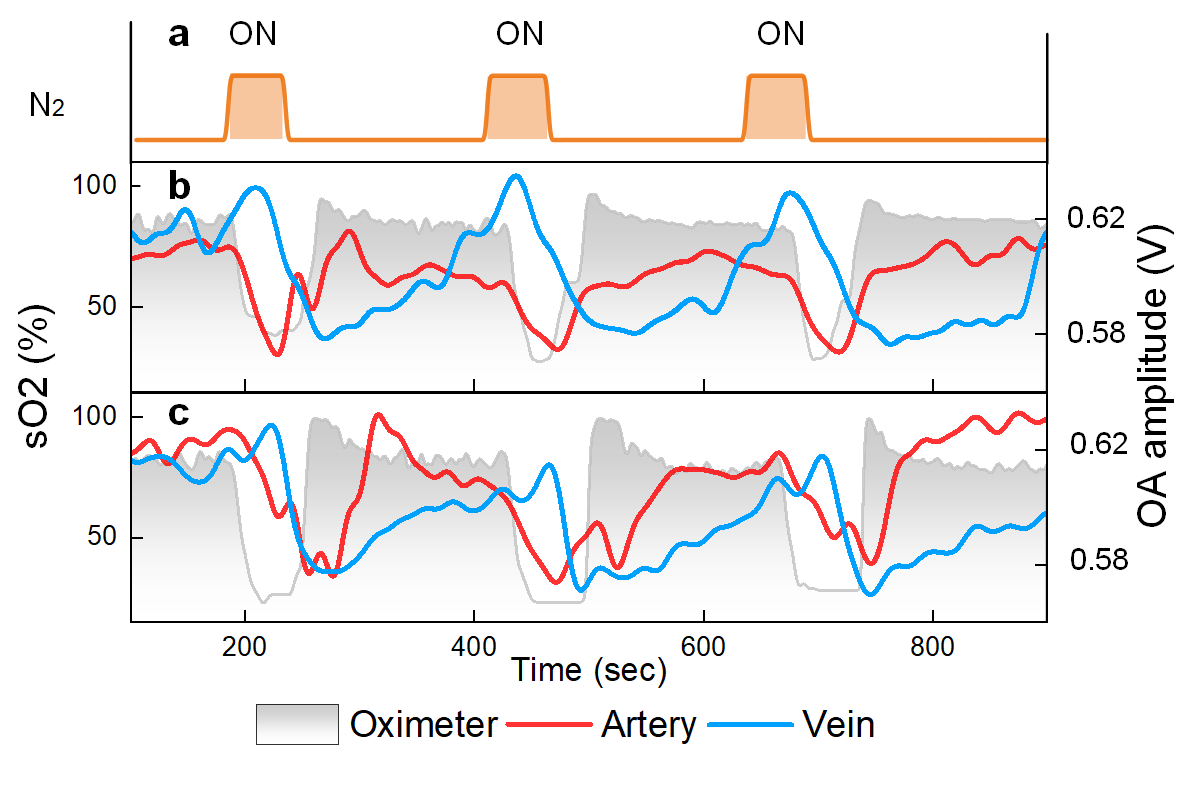


**Supplementary Figure 7. The trend lines of peak-to-peak values of the OA signals measured by OBS in the hypoxia test. a,** Nitrogen output status (nitrogen on means low oxygen supply). **b-c ,** The OA amplitudes of vein and arteries in two sets of experiments. Blue and red lines represent the OA amplitudes of vein and arteries, and the grey region is the oxygen saturation measured by oximeter.


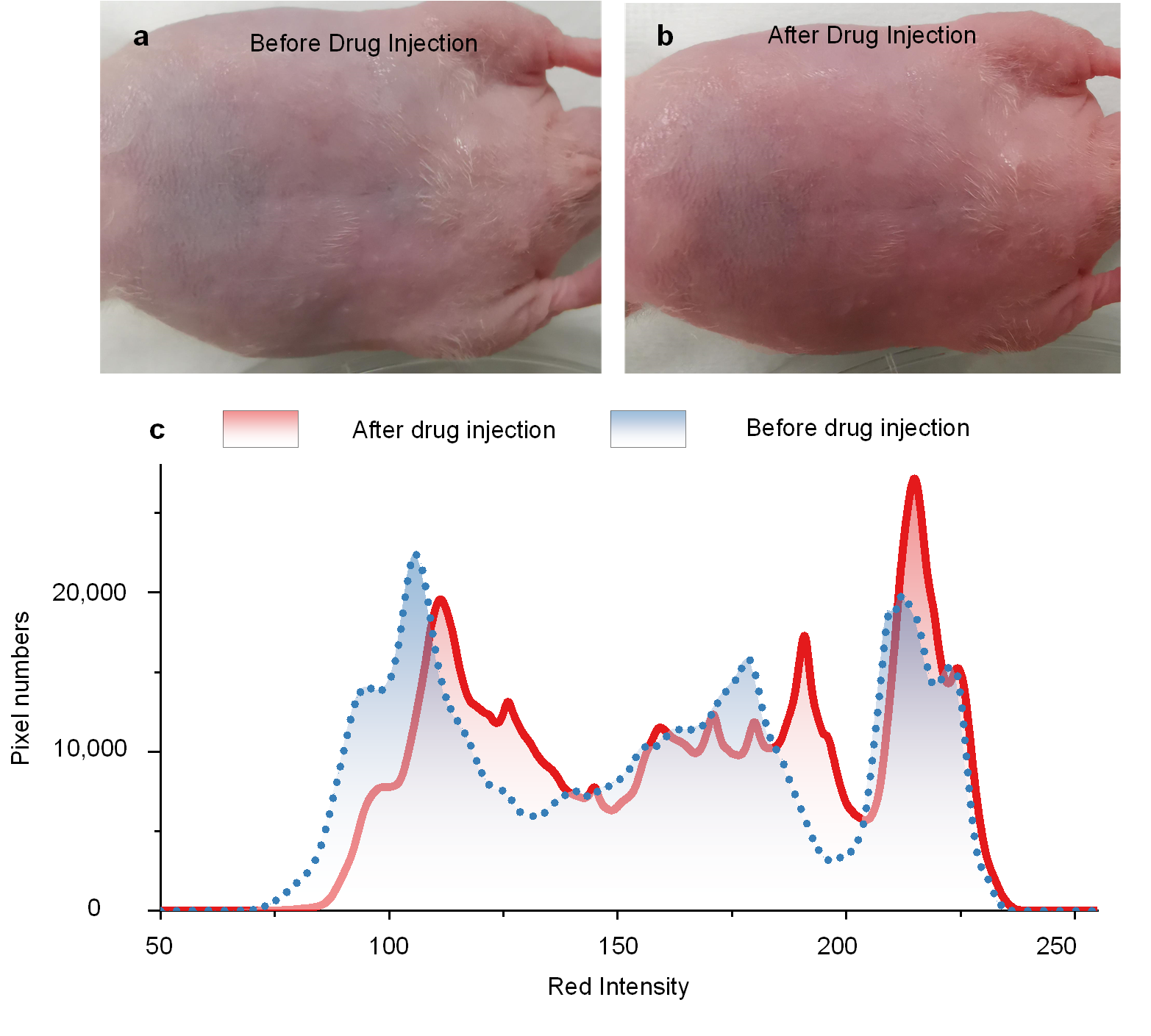


**Supplementary Figure 8. The diffusion of Rhodamine B from blood to tissue. a-b,** Photographs of mice before drug injection (**a**) and after drug injection (**b**). **c,** The histogram of red color intensity in (**a**) and (**b**). After drug injection, the mean red color intensity increases from 143 to 156.


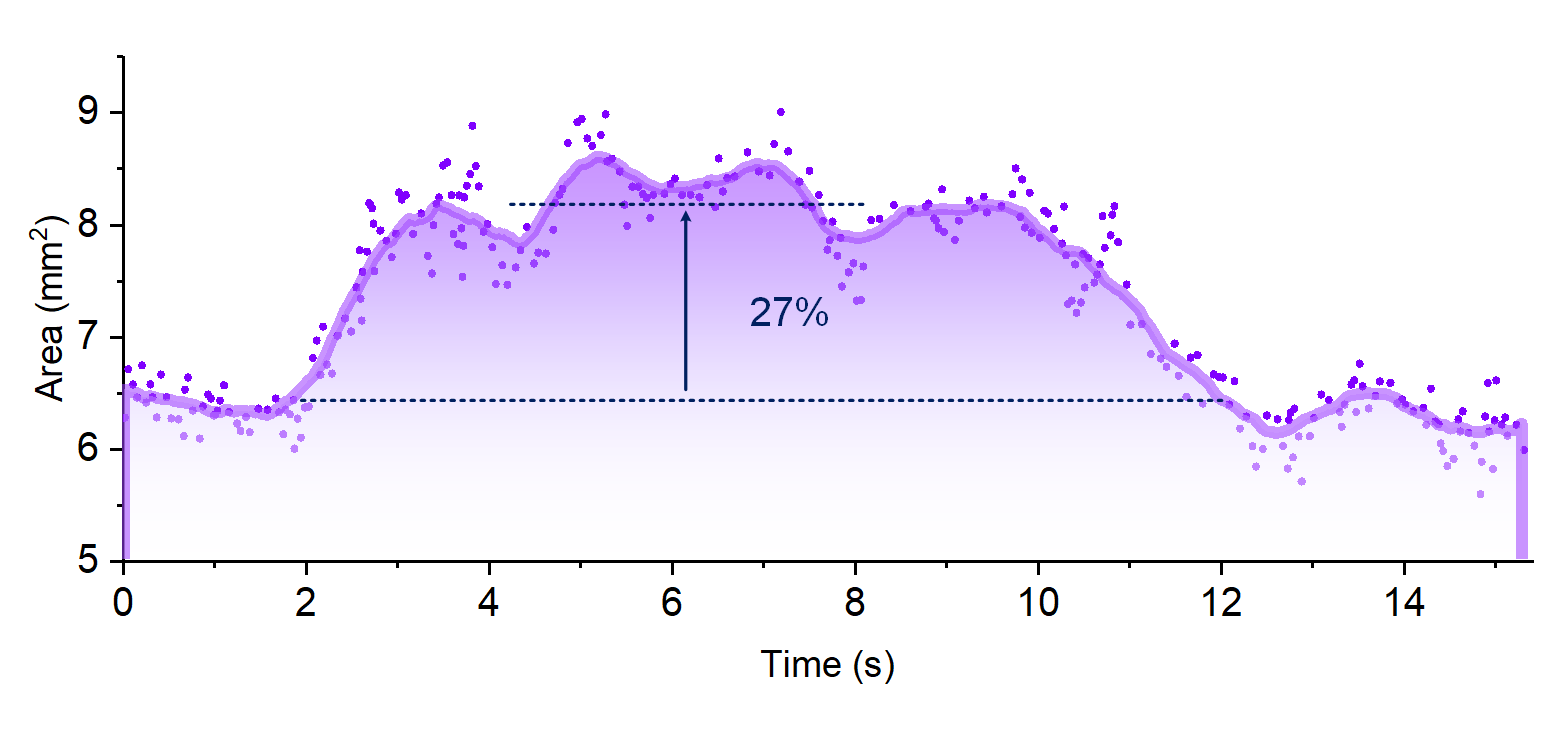


**Supplementary Figure 9. The cross-section area of the dorsal hand vein.** The area increased by 27% under vascular occlusions.


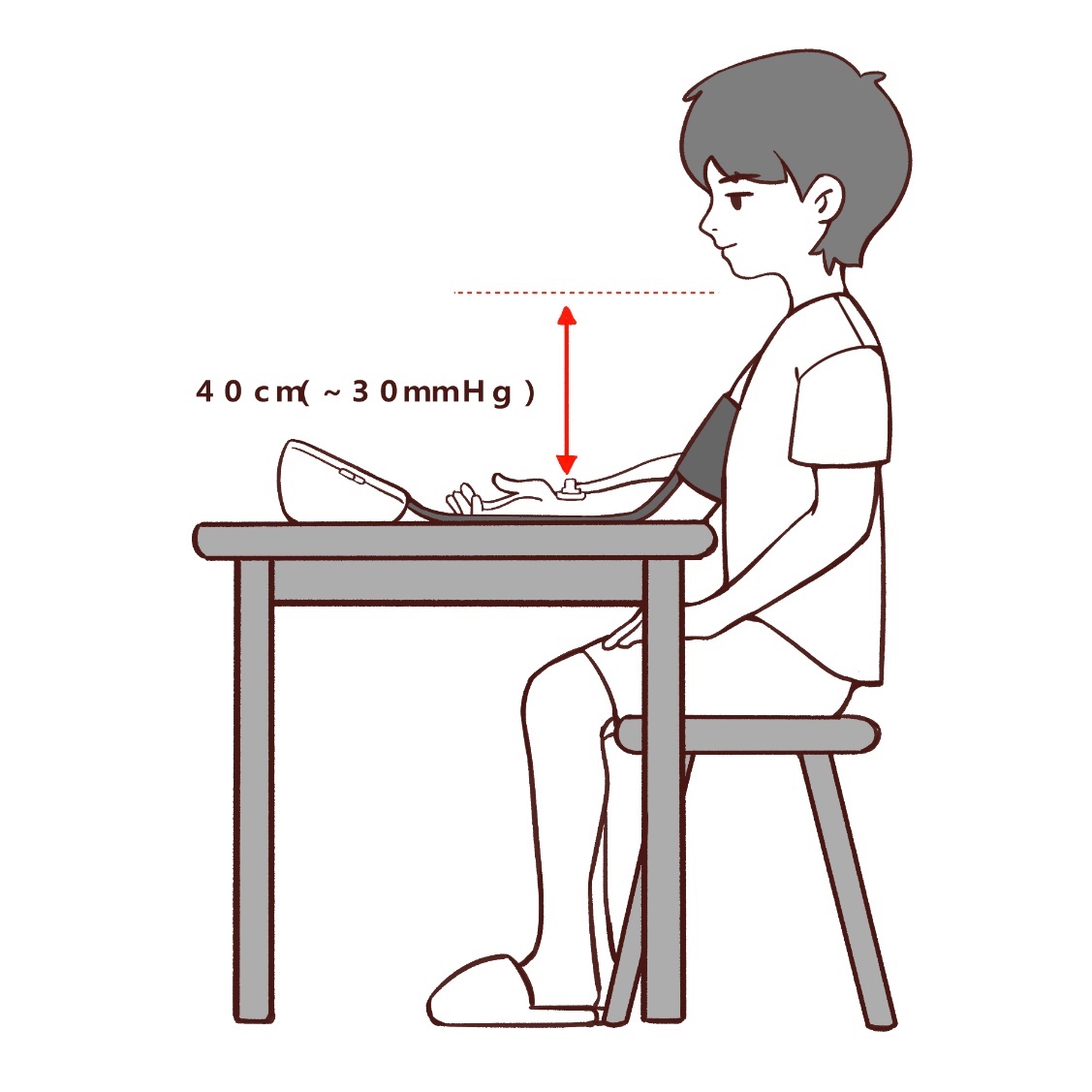


**Supplementary Figure 10. The sitting posture for venous distensibility testing.** The volunteer keeps the height difference between testing points and the neck of 40 cm. In such a posture, the dorsal hand vein's pressure under the cuff releasing condition is about 30 mmHg.

**
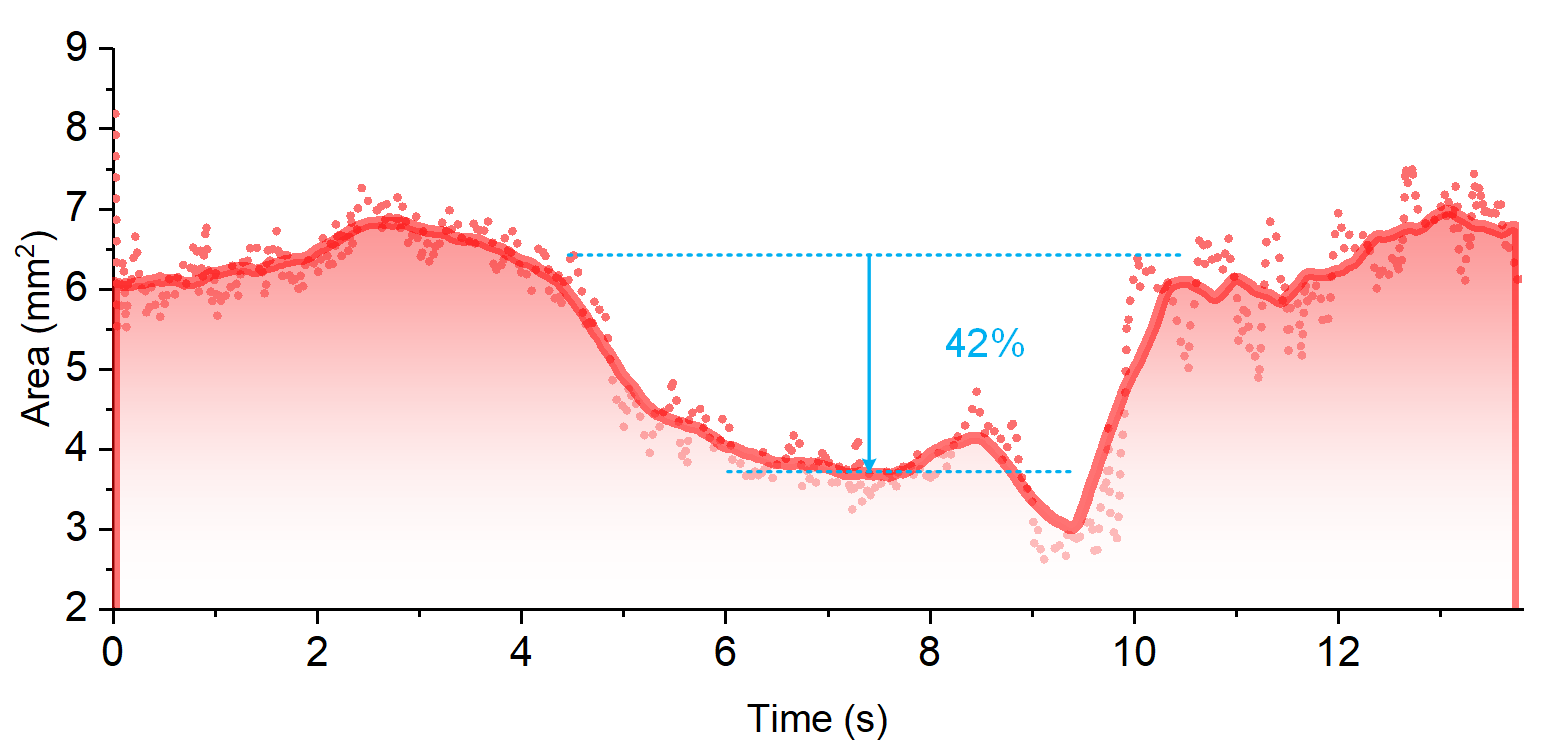
**

**Supplementary Figure 11. The cross-section area of the radial artery.** The area decreased by 42% under vascular occlusions.


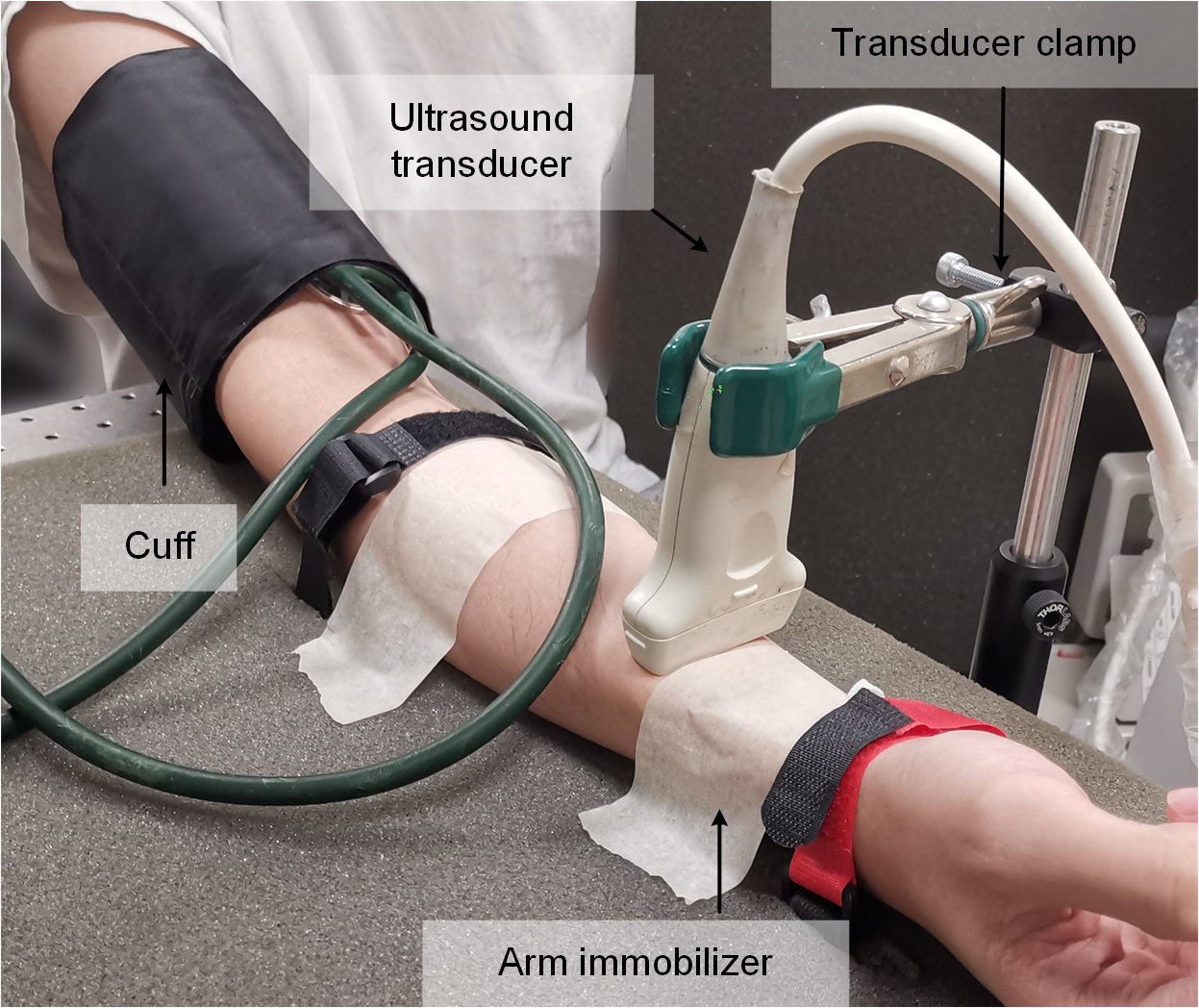


**Supplementary Figure 12. The complicated fixture and process for the FMD tests using traditional ultrasound system.**


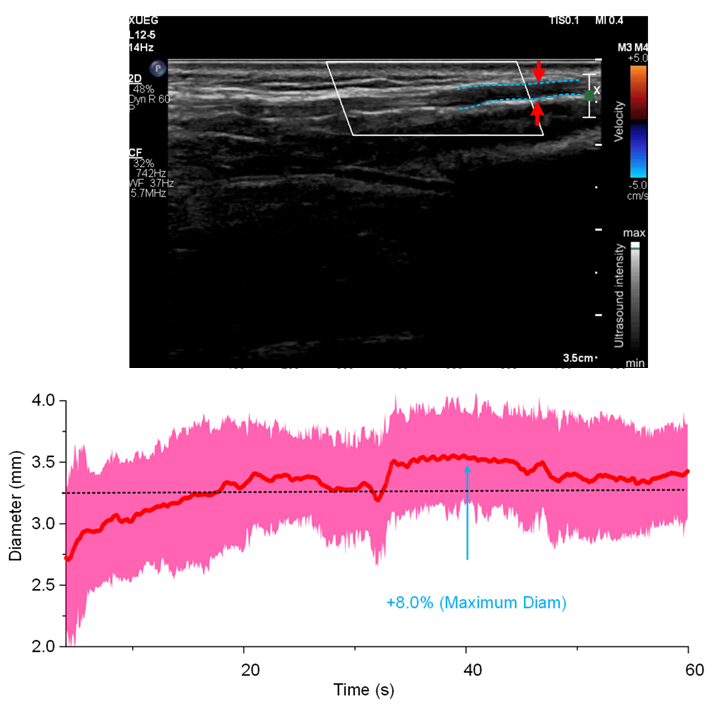


**Supplementary Figure 13. An FMD trail testing with a medical ultrasound system. a,** The snapshot of ultrasound imaging. The blue dot line marked out the radial artery monitored. **b,** The diameter of the radial artery. Its FMD value is about 8.0%. Data are presented as mean values +/- standard deviation.


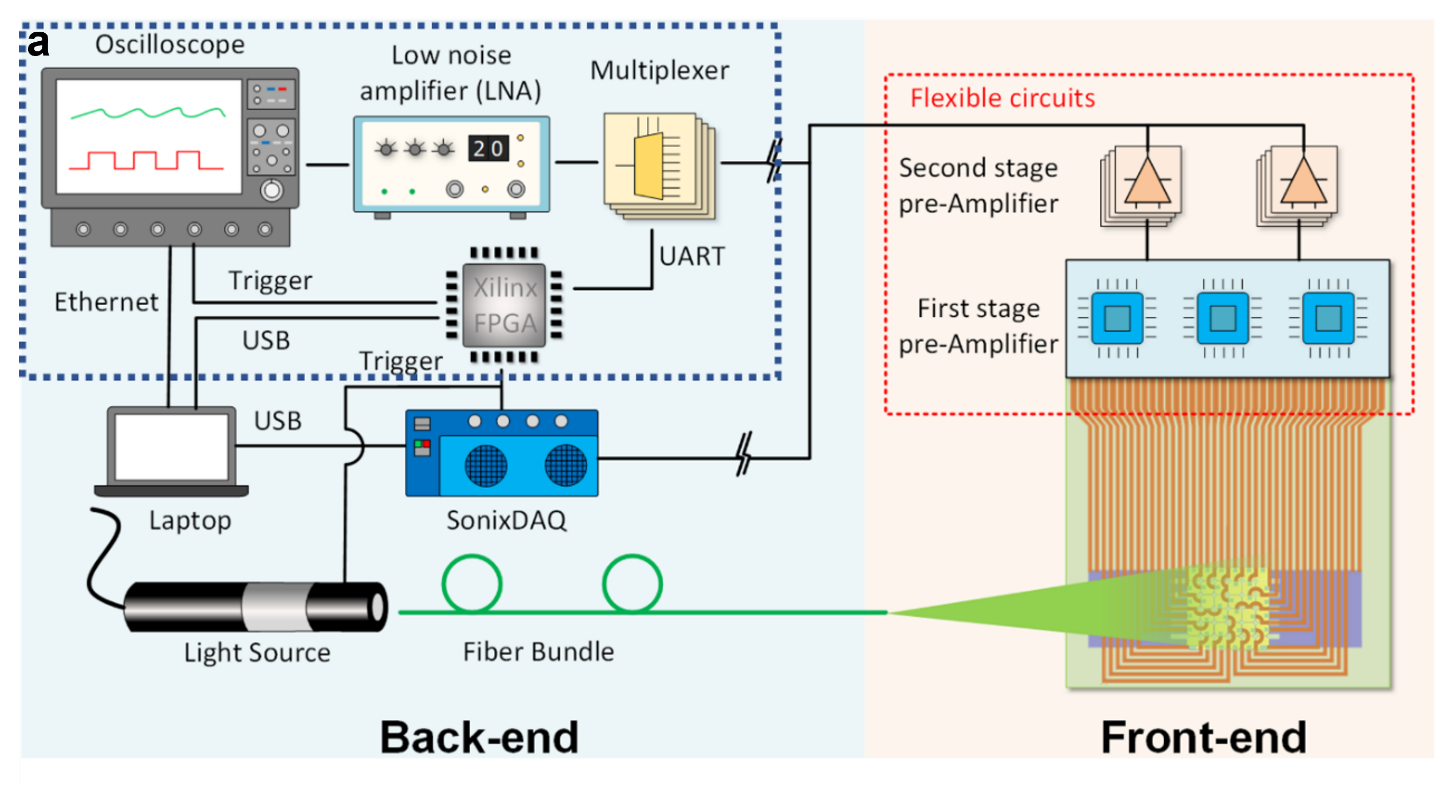


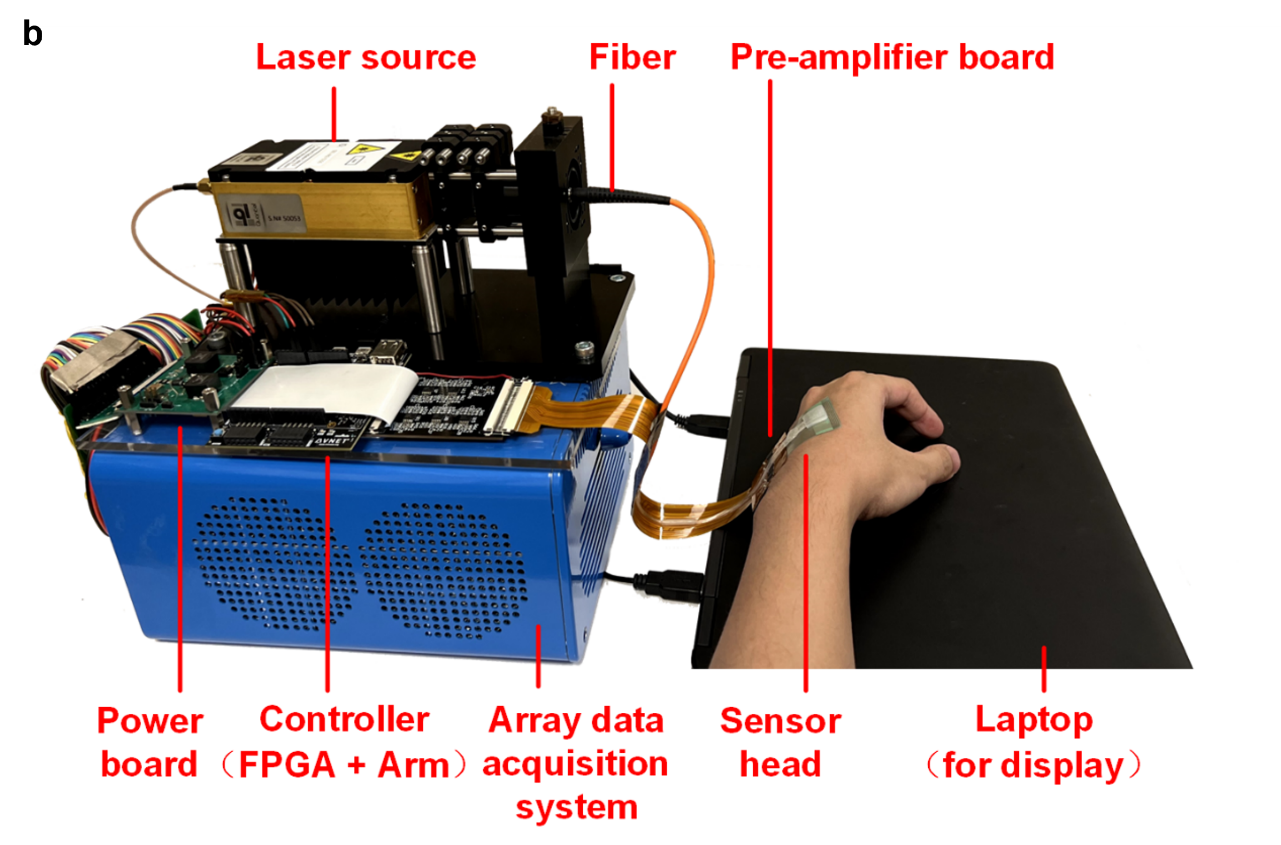


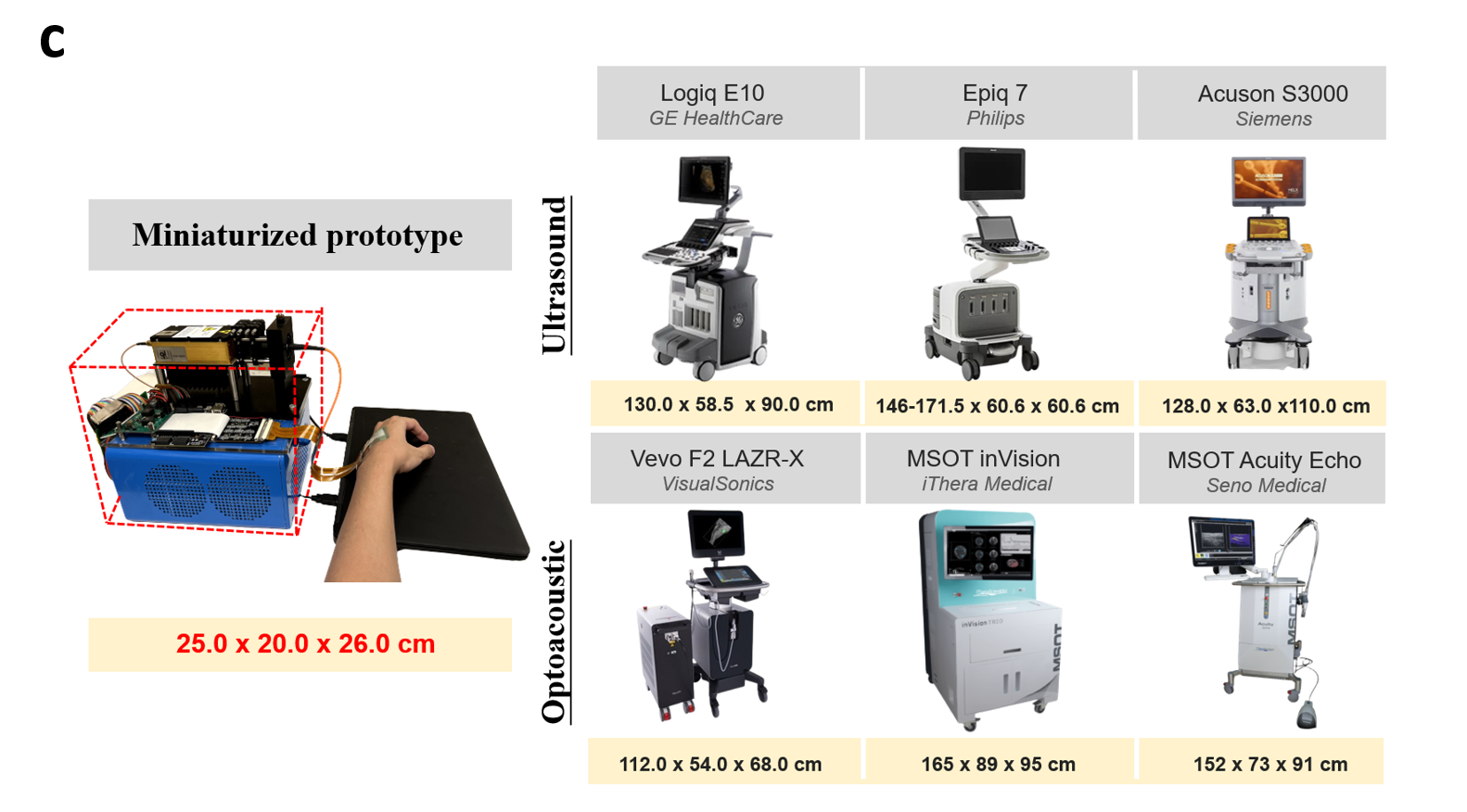


**Supplementary Figure 14. Testing and measurement platform setup. a,** The benchtop system schematic mainly contains front-end and back-end. The front-end is attachable to the skin with the OBS and two-stage pre-amplifier. The back-end includes light supporting, A-scan data acquisition (DAQ) and 3D imaging DAQ systems. The customized 36-channel two-stage pre-amplifiers (MAX4805A, maxim integrated, and TLV3544, Texas Instruments) are responsible for 30 dB pre-amplification. The analogue multiplexers (TMUX1108, Texas Instruments), low-noise amplifier (5072PR, Olympus) and oscilloscope (Lecroy) are responsible for A-scan data acquisition. The multi-channel data acquisition box (SonixDAQ) is for imaging data recording. The light source is generated by pulse laser (Radiant 532LD, Opotek Ltd, UK) and delivered with a customized side output fiber bundle. The whole testing platform is controlled by an FPGA board (Minized, AVENT). **b,** A representative practical implementation towards miniaturization and clinical translation. Compared to the benchtop system schematic, the components enclosed by the upper left blue dashed rectangle have been eliminated from this system's backend, and the bulky benchtop OPO laser has been replaced with a portable dedicated single wavelength laser to reduce overall size. **c,** Comparison of the prototype footprint with those of other commercial ultrasound/optoacoustic systems


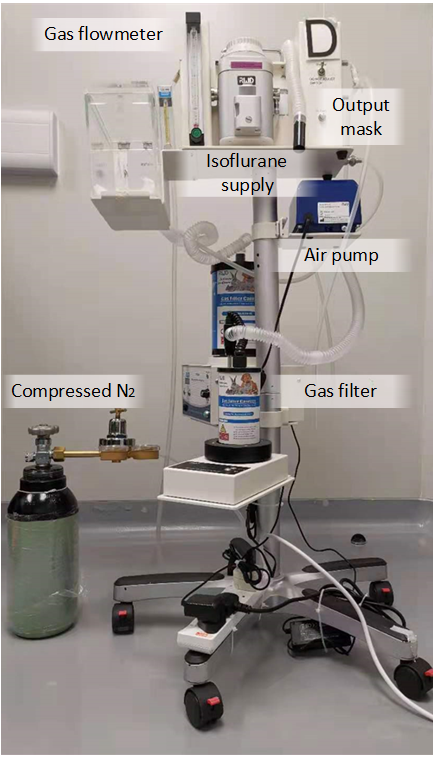


**Supplementary Figure 15. Oxygen and nitrogen gas supply system.**

**Supplementary Notes**

**Supplementary Note 1. Comparison of Ultrasound and Optoacoustic Imaging**

Here, we would highlight the differences between optoacoustic and ultrasonic imaging from the working principles and applications (structural imaging, functional imaging), respectively (Supplementary Figure 16). In terms of working principles, ultrasonic imaging utilizes the transducer to **transmit acoustic waves into media and receive the echoes**, in which the wave reflection only occurs at interfaces of acoustic impedance mismatches [1]. In contrast, optoacoustic imaging **employs laser light to irradiate the tissue and collects the optoacoustic waves generated from the thermal expansion of tissues that absorbs laser energy**. It is undeniable that the ultrasound images can reveal the acoustic impedance mismatches, which contain targets' structure information [2]. **In brief,** structural ultrasound imaging can distinguish **media with** **different mass densities and sound speeds.** As opposed to ultrasonic imaging, structural optoacoustic imaging’s contrast is determined by the **optical absorption spectrum of media**. For instance, variations in blood oxygen saturation have a negligible effect on acoustic impedance (no change in density and sound speed) but can significantly alter the optical absorption spectrum. Essentially, ultrasound imaging is a form of **acoustic imaging modality**, but optoacoustic imaging is more like an **optical imaging modality**. As opposed to structural imaging, functional (physiological) ultrasound imaging can utilize doppler effects to **monitor the directions and velocities of blood flow**. Although optoacoustic can also realize doppler-related functional imaging, it is more mature in adopting multiple wavelengths according to the optical absorption spectrum to **estimate the target’s** **physical and chemical properties**.

When using an ultrasound probe to collect optoacoustic signals, it is often necessary to consider configuring a set of independent light sources, which increases the whole sensor head complexity and is unsuitable for making it flexible and wearable. Inappropriate coupling of acoustic and optical components fails to produce optoacoustic signals and result in laser-induced ultrasound waves on account of the optical absorptive transducers surface, where the absorbed laser energy is converted into the ultrasound waves (Supplementary Figure 17).


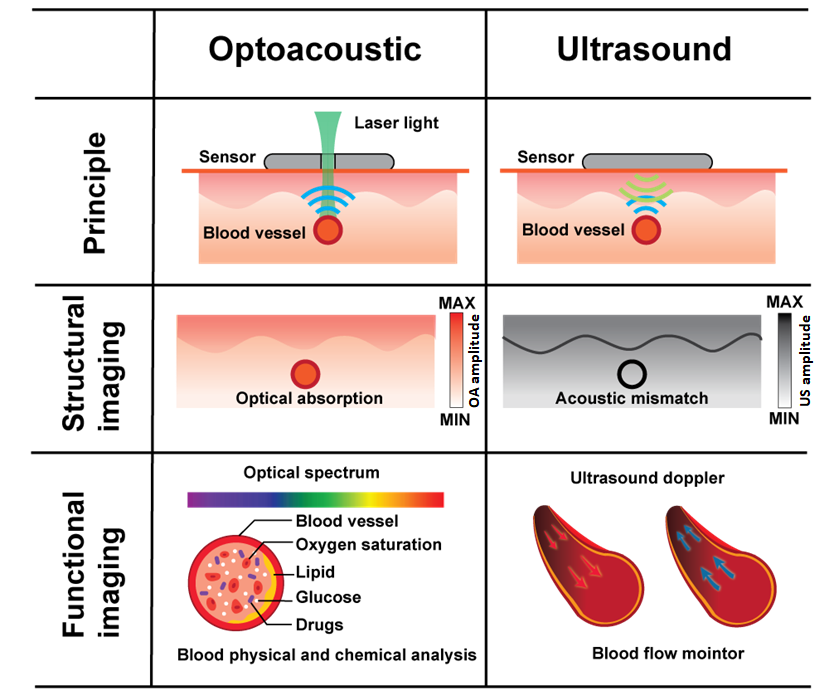


**Supplementary Figure 16. A comparison of optoacoustic and ultrasonic imaging.** **Principle:** optoacoustic imaging received acoustic waves from laser illumination, while ultrasonic imaging utilizes transducer to transmits and receives acoustics waves. **Structural imaging:** structural optoacoustic imaging reveals the optical absorption of media, while structural ultrasonic imaging reflects the acoustic impedance mismatch in media. **Functional imaging:** functional optoacoustic imaging utilizes optical multi-wavelength to indirectly infer blood physical and chemical properties, while functional ultrasonic imaging is able to monitor blood flow by ultrasound doppler.


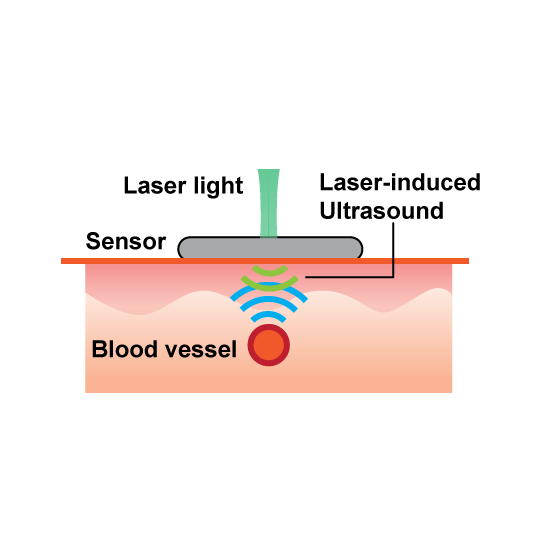


**Supplementary Figure 17. The excitation principle of laser-induced ultrasound waves.**

**Supplementary Note 2. The advantages of the optoacoustic technique over other noninvasive methods.**

OBS utilizes the optoacoustic effect to evaluate the blood vitals and shows several advantages in the following aspects:

1. **3D imaging:** The OBS enables spatially-resolved 3D imaging to clearly differentiate blood characteristics from arteries and veins. On the other hand, the electrochemical/ photoplethysmography/vibrational approaches can only provide an ensemble or point-based statistical reading for the measuring site without resolving any blood vessel morphology. Data mining and artificial intelligence (AI) create greater opportunities to retrieve hidden information from 3D images (with time dimension) than in the time-resolved waveforms.
2. **In situ:** Spatially, the proposed OBS could directly access blood parameters such as hemodynamics from the blood. Conversely, electrochemical techniques can only deduce them indirectly from the body fluid, which is not completely correlated with and may deviate from the actual values.
3. **Real-time:** Temporally, the OBS can acquire blood characteristics in real-time, whereas electrochemical approaches measure body fluids, where there exists a delay in the responding time by referring to reading in body fluids, as the embedded blood biomarkers do not diffuse to the skin instantly.

Supplementary Table 1 gives more comprehensive comparisons of the optoacoustic with other noninvasive techniques commonly used in wearable sensors. Benefiting from the special hybrid imaging modality, the optoacoustic technique empowered OBS can realize rich clinical functional imaging that is different from other wearable techniques. Whereas, photoplethysmography, electrochemical and vibration sensors cannot present vital signals of specific blood vessels, and ultrasound imaging usually provides structural information and limited functional information like hemodynamics using doppler effects.

**Supplementary Table 1. Comparisons of the optoacoustic with other noninvasive techniques**

|  | Photoplethysmography | Electrochemical | Vibration | Ultrasound | **Optoacoustic** |
| --- | --- | --- | --- | --- | --- |
| Related work | [3, 4] | [5-7] | [8] | [9, 10] | [11-13] |
| Potential clinic applications | Rich  (blood oxygen saturation, blood glucose, blood pressure, blood flow) | Rich  (sodium, chloride, potassium, ammonia, lactate, glucose, etc concentration in body fluid) | Poor  (blood pressure, human pulse) | Medium  (hemodynamics) | **Rich**  **(blood oxygen saturation, blood glucose, blood temperature, hemodynamics)** |
| Measurement depth | Poor | Poor | Good | Good | **Medium** |
| Real-time | Yes | No | Yes | Yes | **Yes** |
| 3D imaging capability | No | No | No | Yes | **Yes** |
| Imaging details | NA | NA | NA | Medium | **Good** |

**Supplementary Note 3. Bespoke flexible fiber-based solutions for light delivery.**

In response to the need to accommodate the skin bending for continuous daily monitoring, we have tailored two fiber-based solutions that do not compromise the device's flexibility and wearing comfort. The first implementation, as shown in Supplementary Figure 18(a), is the side-firing encapsulated linear planner bundle, consisting of 5*5 angle-cleaved multimode fibers in alignment with the microlens elements. As illustrated in Supplementary Figure 18(b), the bevel cut at the fiber end (the other side-firing implementations are also applicable) redirects the majority of the fiber axial illumination to the sensor plane normal [14]. Moreover, the strayed light leakage can be further collected and guided towards the skin with the silver reflective coating at the lens cavities (Supplementary Figure 18(c)). Overall, this solution can achieve ~80% side-emitting efficiency. Such a planner layout and flexible supporting encapsulation (Ecoflex™) offer an excellent bending capability, empowering reliable accommodation ability to the skin bending.


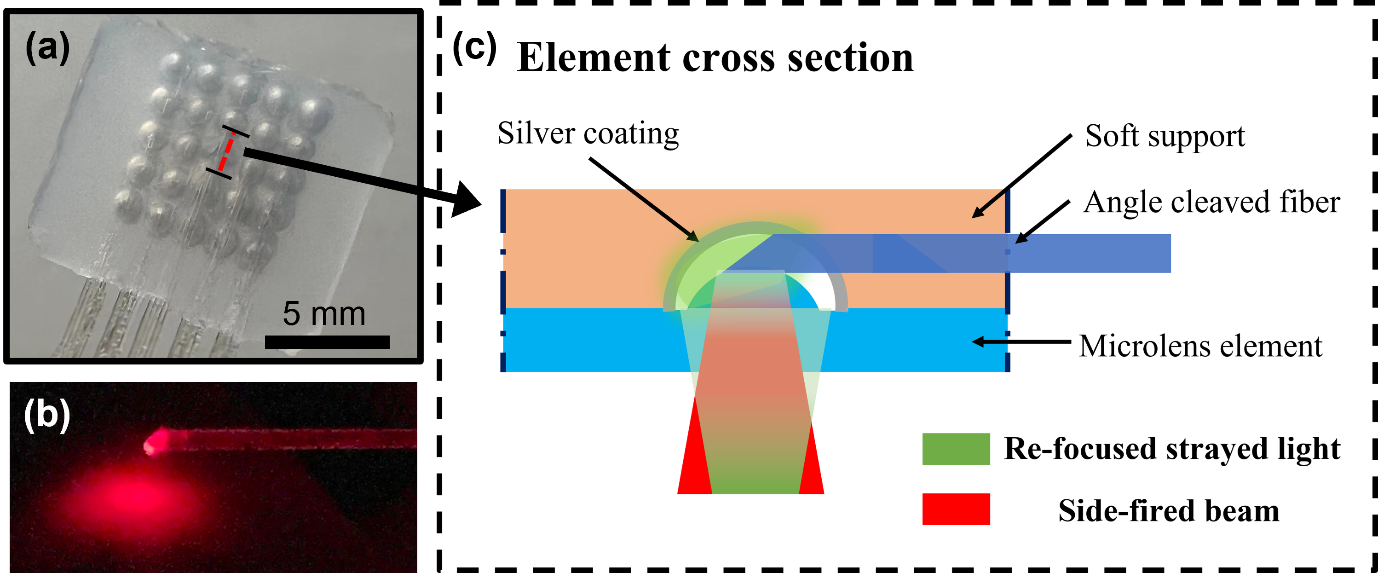


**Supplementary Figure 18.** **The side-firing encapsulated linear planner bundle solution** **(a)** The side-firing encapsulated linear planner bundle. **(b)** Demonstration of side-firing illumination from with single fiber. **(c)** Working principle of the flexible side-firing solution.

Photothermal Therapy (PTT) presents a promising direction for cancer treatment. This process involves administering photothermal agents(intravascular exogenous agents) that absorb light and convert it into localized hyperthermia through nonradiative transitions, leading to tumor ablation. One significant challenge with PTT is real-time, accurate monitoring of the photothermal agent's concentration and localization in the target tissue. These factors can significantly impact the effectiveness of the treatment and potential side effects. Traditional optical methods such as fluorescence imaging and spectroscopy, commonly used for monitoring agent distribution, face limitations due to shallow tissue penetration and signal interference from surrounding tissues.

To overcome these challenges, we employ optoacoustic imaging in our approach. The intravascular exogenous agents can be designed to absorb light at specific wavelengths that penetrate deeper into tissues. The resulting optoacoustic signals facilitate real-time detection and quantification of the photothermal agent's concentration and distribution, providing vital information for optimizing treatment parameters and minimizing side effects.

The continuous monitoring capability of the proposed solution was verified in the following experiment for monitoring the accumulation of photothermal agents via blood circulation in the mice tumor region for 24 hours. Some photothermal agents [15, 16] exhibiting strong optoacoustic response have been applied in photoacoustic (PA) imaging coupled with PTT owing to the high absorption coefficient, good photostability, and suitable biocompatibility. In this experiment, 4T1 tumor-bearing mice were intravenously injected with semiconducting nanoparticles (SCP, PA/PTT agents) at a 10 mg/kg dose, the OBS was attached to the tumor region and PA images were acquired at various time points during the 24 hours, as shown in Supplementary Figure 19. The excellent circulation capability of SCP in mouse blood facilitates their accumulation in the tumor region. The signals increased over time and reached maxima at around 9 h post-injection as shown in Supplementary Figure 20. The remarkable consistency between results obtained with OBS and our previously reported study (with a conventional bulky PA microscopy that required a complex imaging procedure) indicates the reliable continuous monitoring capability of the OBS integrated with the flexible planar light delivery solution.


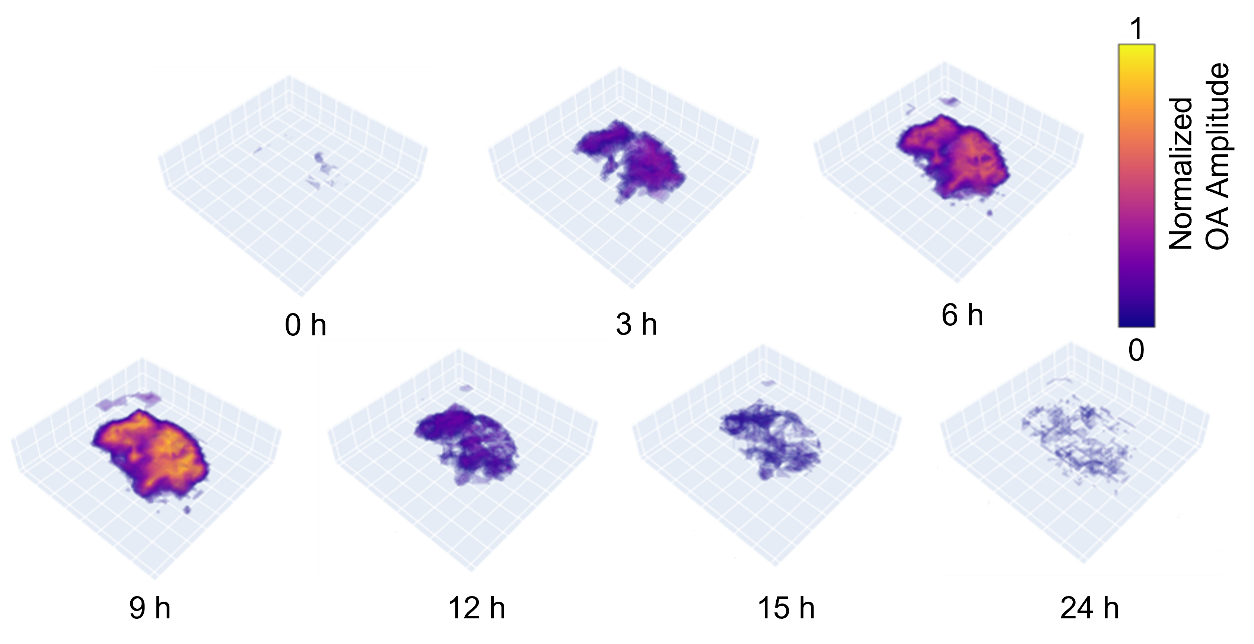


**Supplementary Figure 19. PA images of the tumor region after SCP intravenous administration obtained using our flexible encapsulated planar light delivery solution**


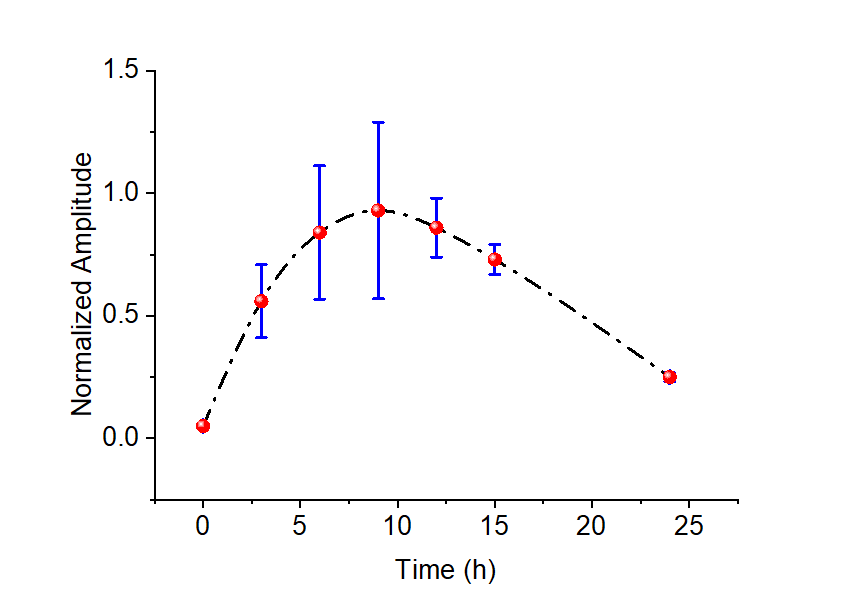


**Supplementary Figure 20. Evolution of PA amplitudes after intravenous administration ( n = 64 independent sampling points, data are presented as mean values +/- standard deviation)**

When higher light flux and better illumination homogeneity are in demand, a multimode round fiber bundle solution with a distal reflector can be an alternative, as shown in Supplementary Figure 21 Both the circular stack fiber bundle and the parabolic reflector are encased with flexible PVC. Although it cannot bend as well as the previous approach due to its greater effective thickness, this flexible implementation nevertheless ensures secure attachment to the region of interest without compromising the wearability. This solution offers robust side-firing directivity and homogeneity with the distal collimating parabolic reflector, in addition to a substantially higher fluence owing to a larger effective fiber core. The feasibility of this solution has been demonstrated by the short-term (from a few seconds to over 30 minutes) experiments presented in the Results section.

**
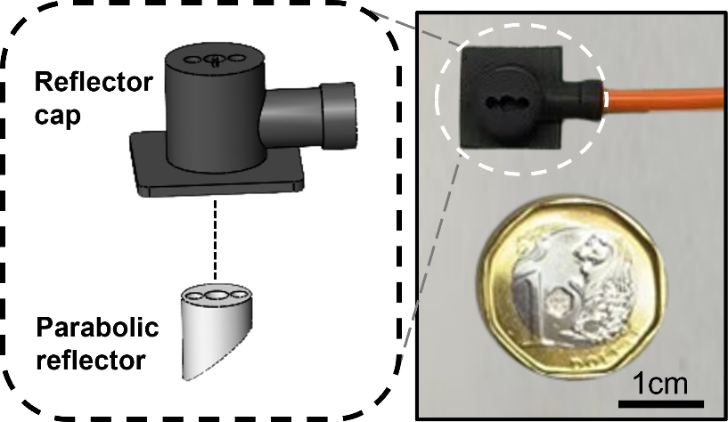
**

**Supplementary Figure 21. The multimode round fiber bundle solution with an exploded view of the distal reflector cap.**

**Supplementary Note 4. Light Delivery Efficiency.**

The illumination source for light delivery efficiency test is a 532 nm pulse laser with an energy of 2 mJ. A powermeter (Gentec-EO MAESTRO, Canada) is used to record the energy of light that penetrates through the sensor head, as shown in Supplementary Figure 22. All the micro-lens layer patched on the sensor are with a 1.6 mm diameter for each lens, while their PDMS thickness is 650 µm, 500 µm, 350 µm, 250 µm, 200 µm and 150 µm, respectively. Also, six flat PDMS films (no micro-lens) with the corresponding thicknesses are also tested as control groups. The tested results are listed in Supplementary Table 2. From the results, the micro-lens could significantly improve the light delivery efficiency, and a thicker layer of micro-lens can obtain higher efficiency. The main reason is a thicker layer provides a longer light path for laser focusing. For the micro-lens layer thicker than 500 µm, the improvement of light delivery efficiency tends to be slow, but the flexibility of the device is severely weakened. Therefore, the best selection of the PDMS thickness is less than 300-500 µm. Meanwhile, the flat absorption spectrum of PDMS indicates the low variations in the transmission coefficients of the OBS at different optical wavelengths (< 3%) [17].

**
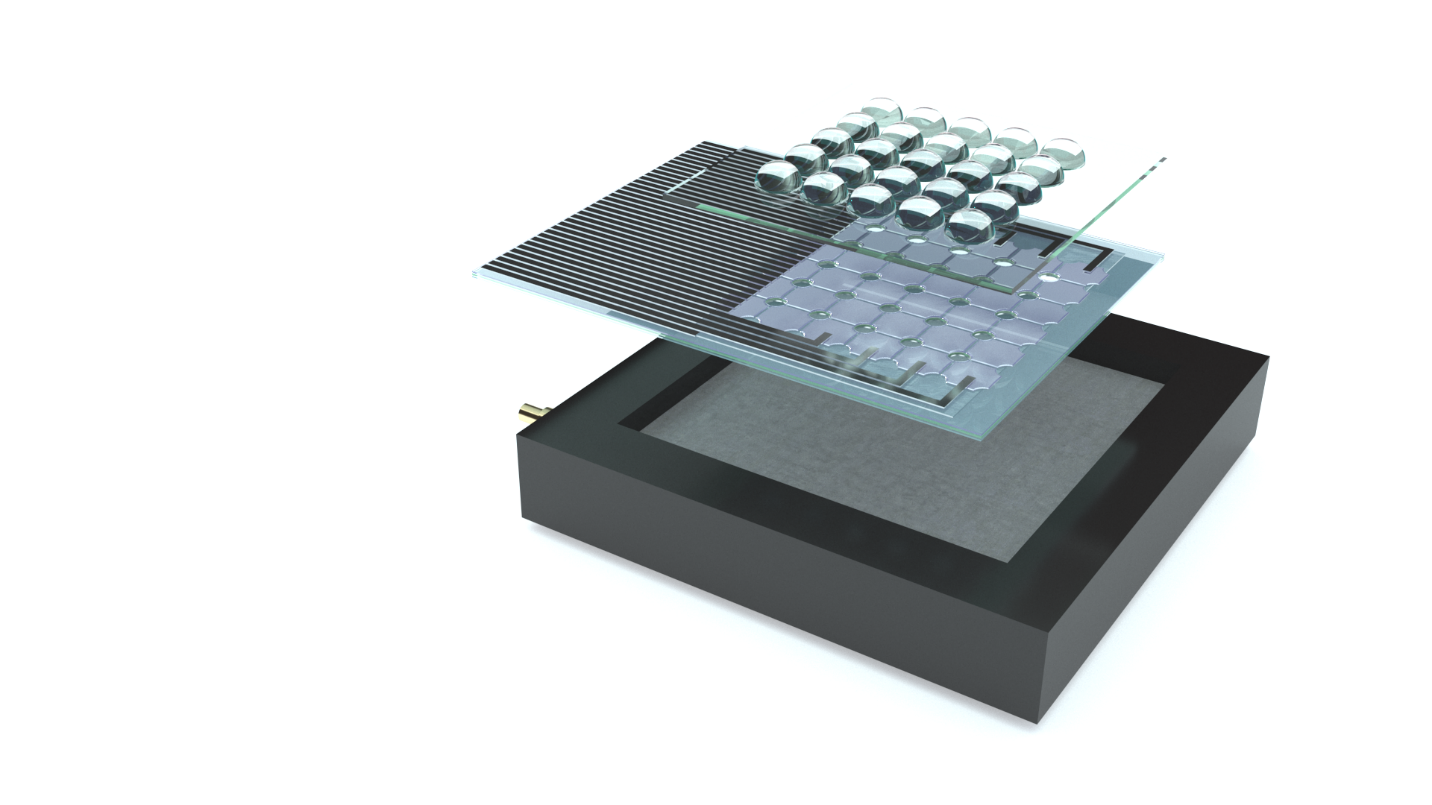
**

**Supplementary Figure 22. The schematic of light delivery testing with powermeter.**

**Supplementary Table 2. The Light delivery efficiency with different micro-lens**

|  | 650 µm | 500 µm | 350 µm | 250 µm | 200 µm | 150 µm |
| --- | --- | --- | --- | --- | --- | --- |
| Micro-lens | 1.31mJ/ (66%) | 1.29mJ /(65%) | 1.20mJ/ (60%) | 1.10mJ/ (55%) | 1.03mJ/ (52%) | 0.96mJ/ (48%) |
| No micro-lens | 0.466mJ/ (23.3%) | 0.472mJ/ (23.6%) | 0.48mJ/ (24%) | 0.491mJ/ (24.5%) | 0.492mJ/ (24.6%) | 0.497mJ/ (24.8%) |

**Supplementary Note 5. The comparison of transparent acoustic sensors.**

Supplementary Table 3 provides an intuitive comparison of different transparent acoustic sensor designs in terms of piezo material, transparency, and resistance. In our OBS, the electrodes are made of silver which has high bendability and conductivity. Benefited from the particular design, its transparency can exceed 65%, covering a wide range of wavelengths (400 nm-1100 nm). In [18, 19], PVDF is also used as piezo-material, but ITO electrodes make the connection resistance over 100 order higher than silver. A 400 nm ITO has little advantage of transparency. In [19], the PVDF thickness is only 9 µm, while our OBS can reach 110 µm; note that PVDF also absorbs light. CMUT is a silicon-based transducer, and LiNbO_3_ is rigid; hence, both are unbendable. The transparency advantages of ITO are not obvious, but the drawback of high resistance is unneglectable. According to [20, 21], a large resistance not only causes signal loss in circuit transmission but also constructs an RC low-pass filter with the side effect of the sensor element to cut-off the high-frequency signals to deteriorate the acoustic resolution.

**Supplementary Table 3. Comparison of Different Transparent Acoustic Sensor Designs**

| Piezo-material | Piezo-thickness | Electrodes | Electrodes  Thickness | Transparency | Resistance | Solutions |
| --- | --- | --- | --- | --- | --- | --- |
| PVDF | 110 µm | Ag | 7 µm | 65% (400 nm-1100 nm) | 10^-2^~10^0^ Ω | Our OBS |
| PVDF | 40 µm | ITO | 200 nm | 80% (450 nm ~2000 nm) | 10^2^-10^4^ Ω | [21] |
| PVDF | 9 µm | ITO | 400 nm | 60% (450 nm ~1100 nm) | / | [20] |
| CMUT | 2.5 µm | ITO | 150 nm | 50% (400 nm-1000 nm) | 1 KΩ | [22, 23] |
| CMUT | 3.3 µm | ITO | 300 nm | 70% (500 nm-600 nm)  50% (600 nm-800 nm) | 35-50 Ω/sq | [24] |
| LiNbO3 | 250 µm | ITO | 200 nm | 80% (690 nm~ 970 nm) | / | [25] |

**Supplementary Note 6. Monte Carlo Simulations.**

The software package MXCLab [26] is used to simulate the light fluence distribution under different illumination conditions. The optical absorptions and scattering coefficients of dermis tissues at different wavelengths are referred to [27]. In all simulations, the voxel is defined as a cube with a side length of 0.05 mm, and the whole grid is defined as 213×213×200. For our proposed illumination solution, the light sources are simulated as 25 cones with a cone angle of 43.6 degrees. In the center window method, a light source with the geometry of conical frustum is designed to illuminate the target. The top radius of the conical frustum is 1.6 mm, and its cone angle is also 43.6 degrees. For the side illumination case, the light sources are simulated as two rectangles that are 0.5 mm×10.6 mm in size. The two lights are respectively titled as 45 and -45 degrees to strike into the tissues. A total of 200 million photons are transmitted into the tissues in all three simulations, and results are collected after photons have scattered and been absorbed for 0.5 ns.

**Supplementary Note 7. Light Pattern.**

In order to clearly record the image of the light pattern, here, a continuous 633 nm laser (HRS015B, Thorlabs) is employed as the light source. The laser beam is expanded by concave (LC1582-A, Thorlabs) and convex (LA1608-A, Thorlabs) lens and regularized with a rectangle iris (GCM-5711M, DHC Ltd, China). Then the light is shooting onto our blood stethoscope. A white paper is attached to the sensor back as a screen, and a CCD camera is used (EOS 80D, Canon) to record the light pattern.


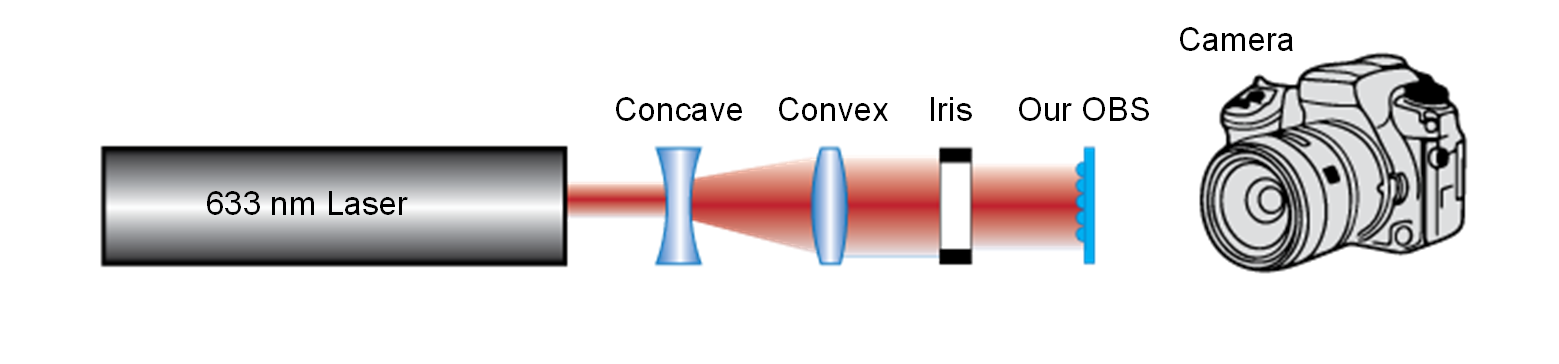


**Supplementary Figure 23. The schematic of light pattern testing with a CCD camera.**

**Supplementary Note 8**. **Light patterns under different bending situations.**

Supplementary Figure 24 shows the light fields under various bending radii ranging from 40 mm to 20 mm in separate columns. Row (b)-(d) show the scattered light patterns behind the probe, with zero, one, and two paper layers at the bottom, respectively. We can conclude that the illumination homogeneity is comparable to the original measurements and not significantly affected by a bending radius within the intended operating range.

It is worth mentioning that besides the OBS, the bespoke side-fire fiber bundle and distal flexible PCB are susceptible to bending. The durability test shows that after 1000 cycles of bending a flexible sensor with a bend radius of 2 mm, no plastic deformation and modification in electrical, optical, or acoustic performance were detected. The minimum bending radius of the latter two components has been retrieved from the datasheet, as listed below.

|  | Flexible amplifier circuit  *(PCBWay)* | Side fire fiber bundles  *(Edmund Optics)* |
| --- | --- | --- |
| Key parameters | Single-sided; polyimide flex substrate; 0.1 mm thickness. | Multimode fibers; buffer layers (Polymide) removed; 100 um core size; 0.22 NA. |
| Minimum bending radius | Short-term: 1 mm  Long-term: 10 mm | Short-term: 11 mm  Long-term: 22 mm |

In conclusion, most of the minimum bending radius values, except the multimode fiber under long-term operation, fall below the minimum desired radius (20 mm), providing additional evidence that the overall device's flexibility and wearing comfort are not impaired during continuous daily operation.

**
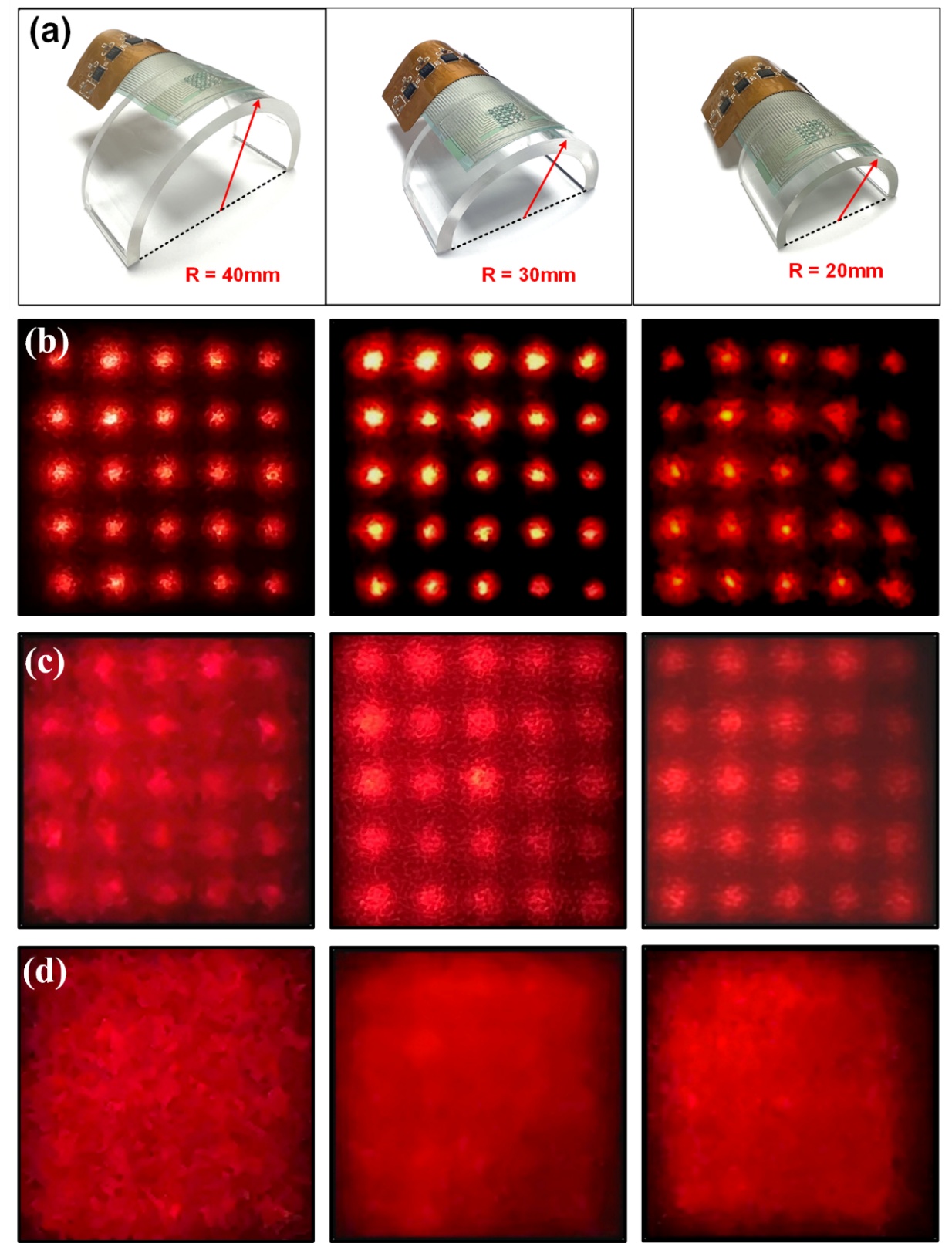
**

**Supplementary Figure 24. Light patterns under different bending situations.** **(a)** Demonstration of the flexibility of OBS and pre-amplifier board and the light patterns **(b)** directly behind the sensor, **(c)** through the sensor with one paper layer at the bottom, and **(d)** with two paper layers at the bottom.

**Supplementary Note 9. Multi-spectral imaging.**

Firstly, the multi-spectral imaging experiment is emulated using black and blue point tapes as targets as shown in Supplementary Figure 25e. A tunable laser (Radiant 532LD, Opotek Ltd), which generates a 5-7 ns duration pulse with 10 Hz repetition frequency in a spectrum ranging from 680 nm to 2600 nm, illuminates the tapes. Four wavelengths of 532 nm, 750 nm, 808 nm, and 1064 nm are used to excite the optoacoustic signals with average energies of 2 mJ, 6 mJ, 6 mJ and 6.5 mJ per pulse, respectively. Our designed devices detect the optoacoustic signals, and signals are recorded by the data acquisition system. The final interpolated images are plotted in Supplementary Figure 25a-d, where two tapes are expressed as two cylindrical scatter clusters. A larger cluster represents a stronger optoacoustic source. According to [28], the amplitudes of optoacoustic waves are determined by light fluence, heat conversion efficiency, optical absorptions, and Grueneisen parameters of targets. In our case, both two tapes are made of PVC (the same Grueneisen and heat conversion efficiency) but with different color (different optical absorption spectra); thus, the amplitudes of received signals should be linearly proportional to the product of light fluence and absorption spectra. As the absolute laser energies vary in different wavelengths, we extracted the amplitude ratios between two tapes under different wavelengths. We then compared the ratio with the absorption spectra results by UV-1800 UV/Visible scanning spectrophotometer (Shimadzu Ltd) as shown in Supplementary Figure 25f. The measured ratios under these four wavelengths are almost the same as those calculated from the absorption spectra in Supplementary Figure 25g. The differences between ratios are mainly related to the inconsistency of light fluences and acoustic sensitivity.


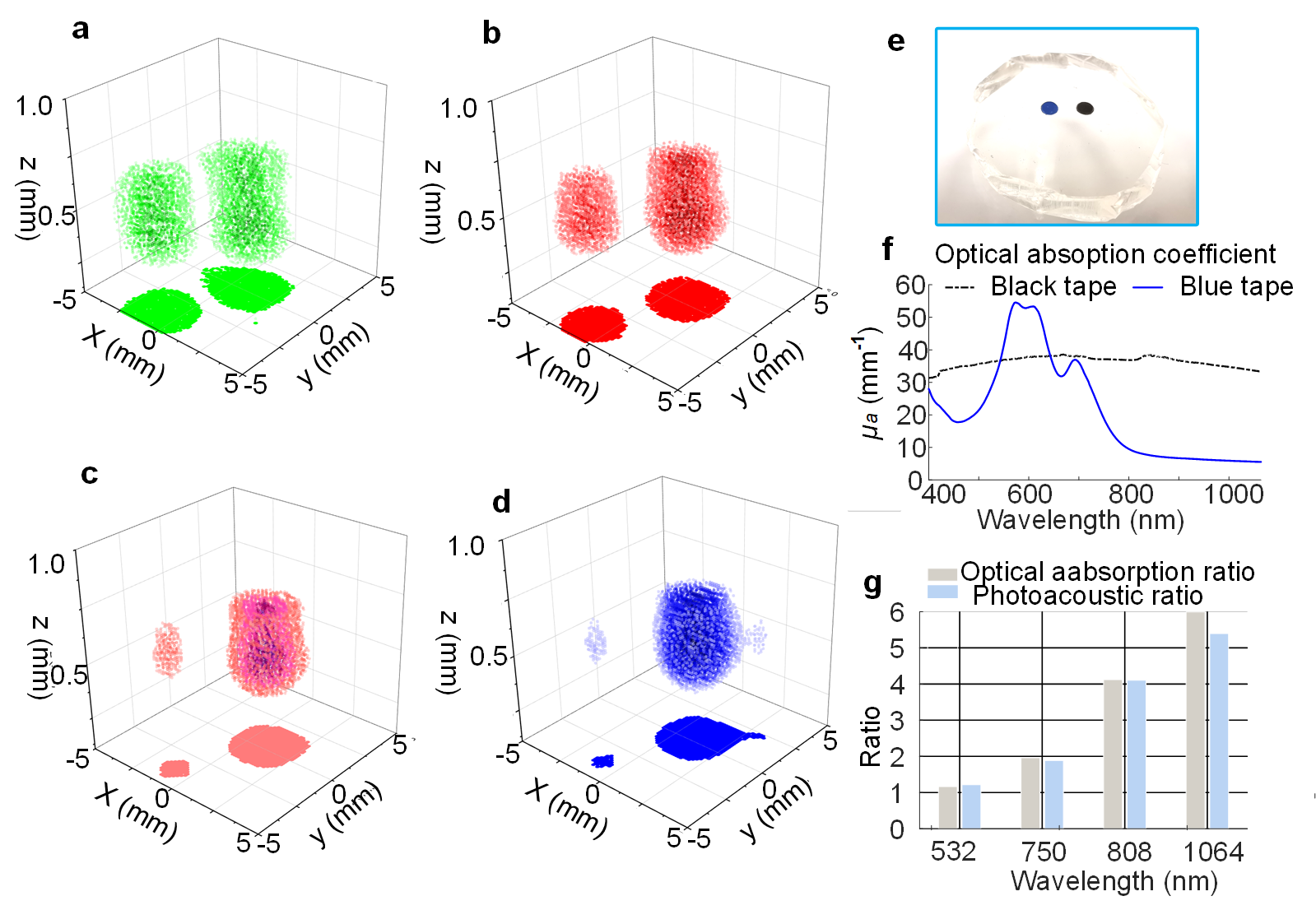


**Supplementary Figure 25. Multi-spectrum imaging on phantom. a,** Photoacoustic imaging using 532 nm laser light. **b,** Photoacoustic imaging using 750 nm laser light. **c,** Photoacoustic imaging using 808 nm laser light. **d,** Photoacoustic imaging using 1064 nm laser light. **e,** Photograph of phantoms. **f,** The optical absorption coefficients of two tapes. **g,** A comparison of optical absorption and optoacoustic amplitude ratios.

**Supplementary Note 10. Mason’s model.**

Mason’s model is a convenient tool to estimate the impedance of PVDF sensors [29]. Supplementary Figure 26 is a Mason’s model of the OBS device, including the front layer, the rear layer, and the acoustic loads. In our case, both the rear layer and the front layer are multilayer structures. According to [30], the *Z_A_* and *Z_C_* of front and rear layers, as well as PVDF and *Z_R_* of acoustic loads, can be calculated by

$$h={e_{33}}/{\varepsilon^{S}},C_{o}=\varepsilon^{S}\frac{A}{d},$$

$$\phi=hC_{o},C=C_{o}\left( 1 - j \phi\right),$$

$$\gamma=j\left( \omega/v \right)\left( 1 - j \Psi/2 \right),$$

$$B=j\rho vA\left( 1 + j \Psi/2 \right),$$

$$Z_{A}=-jB\tanh\left( \gamma d/2 \right),$$

$$Z_{C}=-jB\mathrm{cosech}\left( \gamma d \right),$$

$Z_{R}=\rho v.$ (1)

where $\varepsilon^{S}$ is the clamped dielectric constant, $e_{33}$ is the piezoelectric stress constant, $v$ is the acoustic velocity of material, $\omega$ is the radian frequency, $\rho$ is the density of the material, $A$ is the area of the material, $d$ is the thickness of the material, $\Psi$ is equal to the mechanical loss tangent, $C_{o}$ is the static capacitance of piezoelectric material. The acoustic impedance of the OBS can then be obtained by a calculation of series-parallel impedance following Supplementary Figure 27. The detailed parameters of the PVDF and other materials are listed in Supplementary Table 4 and Supplementary Table 5, respectively. It can be seen that increasing the thickness of PVDF and Ag electrodes will drop the center frequency and vice versa.

**Supplementary Figure 26. Mason’s model including front and rear layers and acoustic loads.**

**Supplementary Table 4. PVDF parameters**

| Parameters | PVDF |
| --- | --- |
| $\rho$(kg/m^3^) | 1780 |
| $v$(m/s) | 2200 |
| $e_{33}$ | -0.16 |
| $\Phi$ | 0.17 |
| $\Psi$ | 0.077 |

**Supplementary Table 5. Densities and sound speeds of materials**

| Material | $\rho$(kg/m^3^) | $v$(m/s) |
| --- | --- | --- |
| PDMS | 969 | 1119 |
| Ag | 10530 | 3600 |
| Cu | 8900 | 4720 |
| Cr | 7140 | 5940 |
| Air | 1.3 | 340 |

**
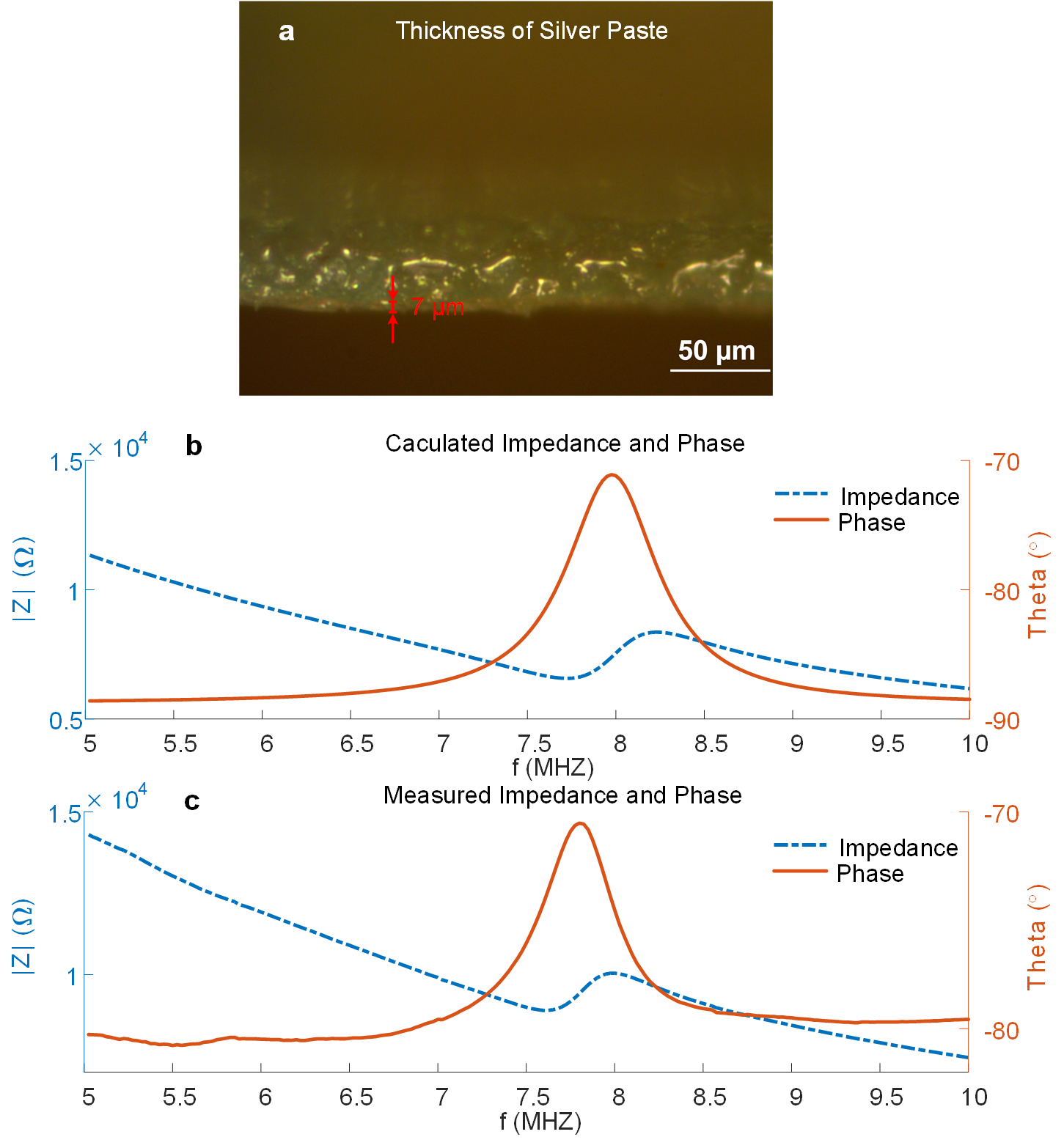
**

**Supplementary Figure 27. Impedance analysis with Mason’s model. a,** The photograph of OBS sliver paste layer. **b,** The impedance and phase calculated by Mason’s model. **c,** The impedance and phase of our OBS.

**Supplementary Note 11. Acoustic characterization calculation.**

The axial resolution of the sensor is expressed as

$R_{axial}=0.88\frac{c}{\Delta f}$ (2)

Where $c$ is the speed of sound, and $\Delta f$ is the bandwidth of the sensor.

The acoustic wavefield of a single sensor is calculated with integration as

$p\left( \boldsymbol{r} \right)=\int_{\boldsymbol{S}} \frac{e^{-j\omega\left\| \boldsymbol{r} - \boldsymbol{r}_{\boldsymbol{d}} \right\|/\boldsymbol{c}}}{\left\| \boldsymbol{r} - \boldsymbol{r}_{\boldsymbol{d}} \right\|}d\boldsymbol{r}_{\boldsymbol{d}}$ (3)

where $\boldsymbol{r}$ is the position of the measuring point, and $\boldsymbol{r}_{\boldsymbol{d}}$ represents the position of points inside the sensor. $\boldsymbol{S}$ denotes the surface of the sensor, $\omega$ is the angular frequency. The acoustic field of view (AFOV) of OBS is written by summing the wavefields of all 36 sensors as

$p\left( \boldsymbol{r} \right)=\sum_{k=1}^{36} \left[ \int_{\boldsymbol{S}_{k}} \frac{e^{-j\omega\left\| \boldsymbol{r} - \boldsymbol{r}_{\boldsymbol{d}}^{\left( k \right)} \right\|/\boldsymbol{c}}}{\left\| \boldsymbol{r} - \boldsymbol{r}_{\boldsymbol{d}} \right\|}d\boldsymbol{r}_{\boldsymbol{d}}^{k} \right]$ (4)

where the $\boldsymbol{r}_{\boldsymbol{d}}^{k}$ represents the position of the differential point of sensor $k$, and $\boldsymbol{S}_{k}$ is the integral region of sensor $k$.


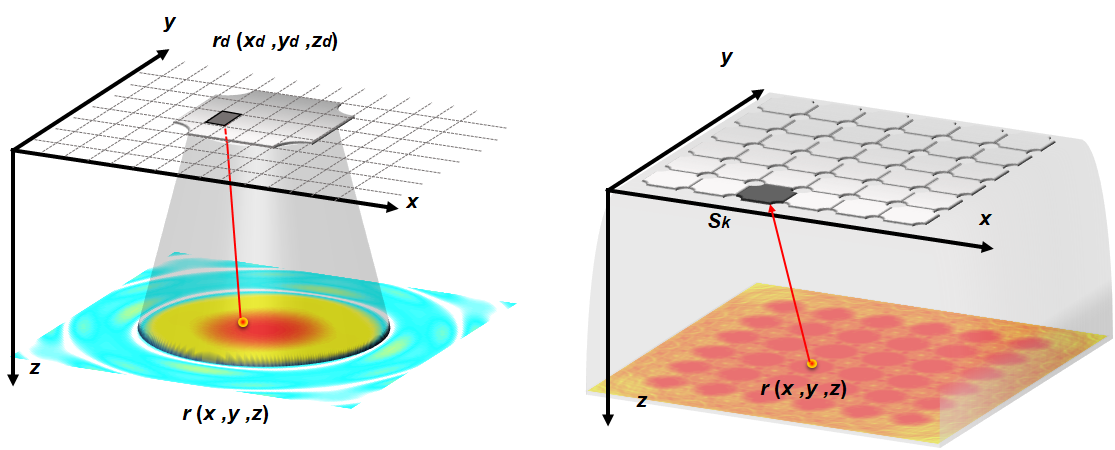


**Supplementary Figure 28. The schematics of the AFOV calculations. a,** The acoustic wavefield of a single element. **b,** The AFOV of 36 acoustic elements.

**Supplementary Note 12. The actual acoustic detection sensitivity characterization**.

To characterize the acoustic field, a simple phantom study is conducted. The protocol for the experiment is described as follows: The characterization target, a black tape, was initially positioned 1 mm from the sensor plane. After focusing on the black surface, a diffraction-limited laser spot was swept across the sensor aperture. Following this procedure, it is possible to collect the detection sensitivity profile represented by OA signal amplitude. The outcomes are depicted in Supplementary Figure 29. The measured effective spatial response region (-12 dB) swiftly exceeds the sensor area of 2.28 mm^2^ and reaches 3.17 mm^2^. Meanwhile, the mean versus maximum acoustic amplitudes from the effective AFOV is about -3.1 dB with an s.d. of 1.4 dB which can be further reduced to s.d. of 1 dB at a depth of 2 mm. Compared with the theoretical results, both the actual detection sensitivity profiles of the single element and the full array are smoother; therefore, the actual acoustic fields are also uniform. It can be seen from Supplementary Figure 29(d) that the variation of detection sensitivity is only -6dB at a depth of 1 mm. Note that, AFOV will be more uniform as the detection depth increases.


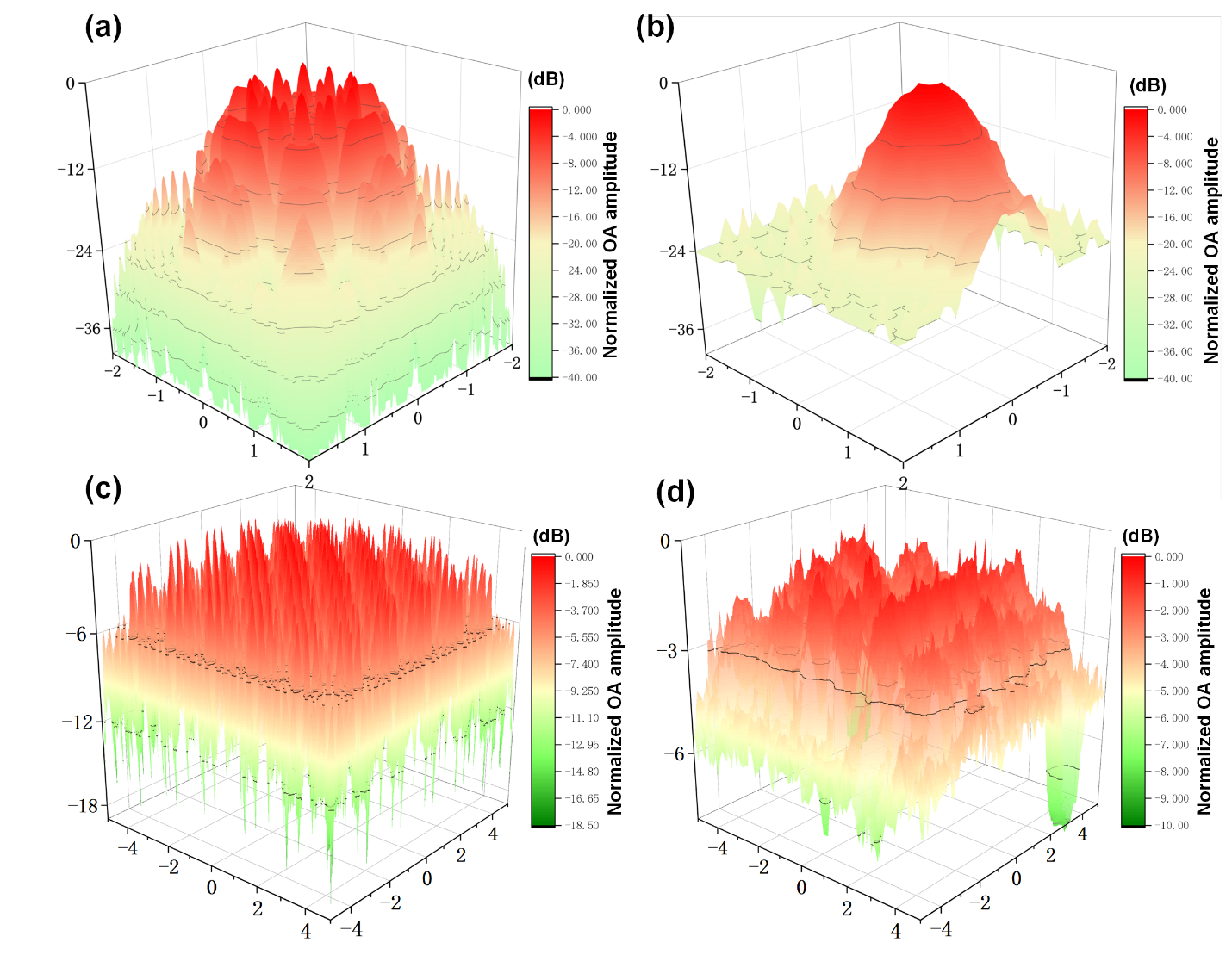


**Supplementary Figure 29. The comparison of theoretical and actual acoustic detection sensitivity.** **(a)** The theoretical detection sensitivity of a single element. **(b)** The measured detection sensitivity of a single element. **(c)** The theoretical detection sensitivity of array sensor. **(d)** The measured detection sensitivity of the array sensor.

**Supplementary Note 13. Calculation of the detection directivity and grating lobe.**

Generally, the directivity function of the area array sensor (Supplementary Figure 30(a)) is defined as follows, according to [31].

$Dir\left( \theta_{1},\theta_{2}|\theta_{s1},\theta_{s2} \right)=\frac{\sin\left( Nkd_{1}(sin \theta_{1}-sin\theta_{s1})/2 \right)}{\mathrm{Nsin} \left( kd_{1}(sin \theta_{1}-sin\theta_{s1})/2 \right)}\frac{\sin\left( Nkd_{2}(sin \theta_{2}-sin\theta_{s2})/2 \right)}{\mathrm{Nsin} \left( kd_{2}(sin \theta_{2}-sin\theta_{s2})/2 \right)}\frac{sin(ka(sin \theta_{1}-sin\theta_{s1})/2)}{(ka(sin \theta_{1}-sin\theta_{s1})/2)}\frac{sin(kw(sin \theta_{2}-sin\theta_{s2})/2)}{(kw(sin \theta_{2}-sin\theta_{s2})/2)}$ (5)

where $\theta_{1}$ and $\theta_{2}$ are directivity angles in the *x* and *y* directions, respectively. Here, $\theta_{s1}$ and $\theta_{s1}$ are the beam steering angles at x and y directions respectively. For the acoustic nodes in our OBS, $a=1.6 mm, w=1.6 mm, d_{1}=1.75 mm, d_{2}=1.75 mm$, and $N=6.$ According to Eq. 5, the ratios of main lobes to grating lobes with different steering angles are all higher than 19 dB. The grating lobes would not cause severely artifacts. The simulation in Supplementary Figure 30(b) shows that the main lobe at steering angle of zero has a significantly greater amplitude than the grating lobes (20 dB higher compared with the first-order grating lobe) at the center frequency of 7.8 MHz ($k=3.3\times{10}^{4}$). Therefore, the impact of grating lobes on the reconstruction is expected to be trivial in our design, and hence should not significantly degrade the imaging quality and resolution.


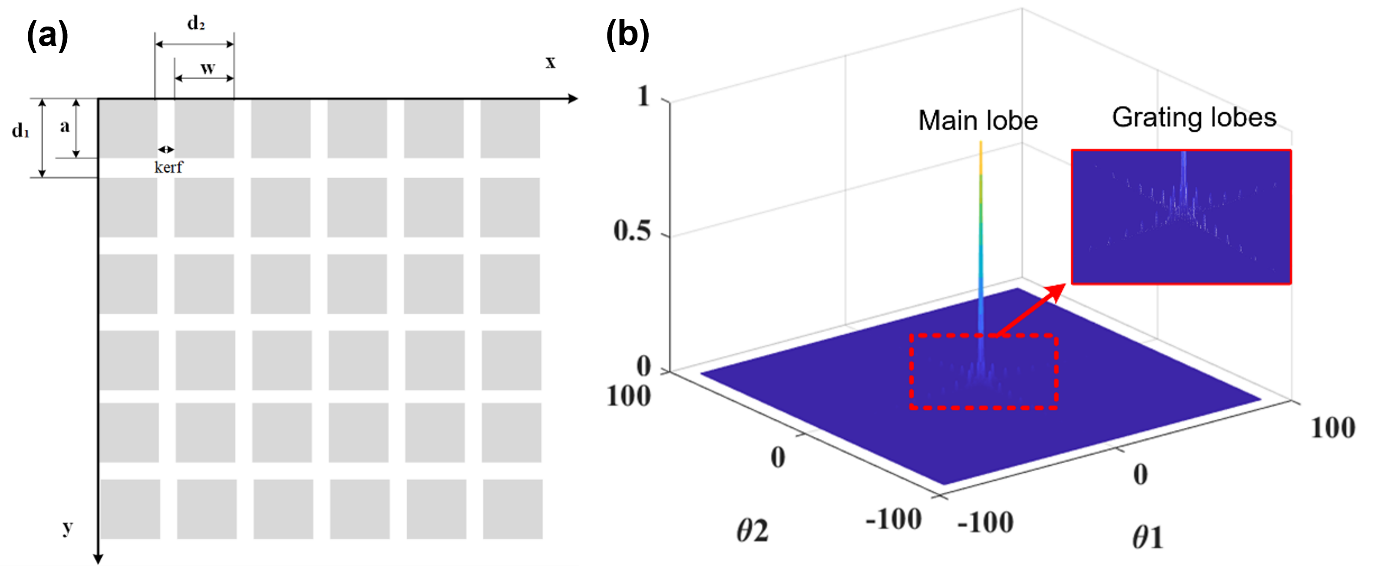


**Supplementary Figure 30. Detection directivity characterization. (a)** The geometry of acoustic nodes. **(b)** The two-dimensional directivity function of acoustic nodes.

**Supplementary Note 14. Derivation of 3D imaging algorithm [32, 33].**

The derivation of PS-NUFFT is established upon the more exact Fourier domain physical modeling. Various acoustic effects like attenuation, dispersion, diffraction, refraction, etc, could be considered to account for heterogeneous media, making PS-NUFFT versatility adaptive for complex optoacoustic imaging scenarios.

The 3D imaging using OBS under a uniform light illumination could be described as a classical acoustic receiving model, as [34]

$\nabla^{2}p\left( x , y , z , t \right)-\frac{1}{c^{2}}\frac{\partial^{2}}{\partial t^{2}}p\left( x , y , z , t \right)=0,$ (6)

subject to the initial conditions

$$p\left( x , y , z , t \right)|_{t=0}=S\left( x , y , z \right),$$

$$\partial p\left( x , y , z , t \right)/\partial t|_{t=0}=0.$$

where $S\left( x , y , z \right)$ is the acoustic source terms which is only excited at the moment of laser irradiation. Assuming the acoustic elements lie on the skins at a depth $z=0$, all sound sources are buried under the skin as $z>0$. Thus, the pressures recorded by the acoustic elements are represented as $p\left( x , y , z = 0 , t \right)$. Therefore, the image reconstruction could be treated as using $p\left( x , y , z = 0 , t \right)$ to solve $S\left( x , y , z \right)$.

Firstly, the recorded pressures data $p\left( x , y , z = 0 , t \right)$ is decomposed into a set of harmonic plane wave components as

$p\left( x , y , z = 0 , t \right)=\iiint P\left( k_{x} , k_{y} , z = 0 , \omega\right)e^{ik_{x}x}e^{ik_{y}y}e^{-i\omega t}dk_{x}dk_{y}d\omega$ (7)

where $k_{x}$ , $k_{y}$ , and $\omega$ represent the wavenumber of $x$, $y$, and $t$, respectively. A phase shift term can be applied to the plane wave component to backpropagate the wavefields to an arbitrary depth $z_{s}$ as

$P\left( k_{x} , k_{y} , z_{s} , \omega\right)=P\left( k_{x} , k_{y} , z = 0 , \omega\right)e^{ik_{z}\left( z_{s} - z \right)},$ (8)

where $k_{z}$ is determined by the $k_{x}$ , $k_{y}$ , and $\omega$ of each wave component with relation as

$\left( \omega/ c \right)^{2}=k_{x}^{2}+k_{y}^{2}+k_{z}^{2}.$ (9)

It is convenient to obtain the source term at depth $z_{s}$ using an inverse Fourier transform on $P\left( k_{x} , k_{y} , z_{s} , \omega\right)$ with a condition $t=0$, as

$S\left( x , y , z_{s} \right)=\iiint P\left( k_{x} , k_{y} , z_{s} , \omega\right)e^{ik_{x}x}e^{ik_{y}y}dk_{x}dk_{y}d\omega$. (10)

However, such a method solves the $S\left( x , y , z \right)$ layer by layer. To improve computational efficiency, an integral transform is introduced to modify Eq.9. By substituting Eq. 7 into Eq.9, a general form of backpropagation is written as

$S\left( x , y , z \right)=\iiint P\left( k_{x} , k_{y} , z = 0 , \omega\right)e^{ik_{x}x}e^{ik_{y}y}e^{ik_{z}z}dk_{x}dk_{y}d\omega.$ (11)

According to Eq.10, the derivative of $\omega$ is expressed as

$d\omega=ck_{z}dk_{z}/\sqrt{k_{x}^{2}+k_{y}^{2}+k_{z}^{2}}.$ (12)

Substituting Eq.11 into Eq.10, the backpropagation is rewritten by transforming the integral from $\omega$ domain to $k_{z}$ domain,

$S\left( x , y , z \right)=\iiint P\left( k_{x} , k_{y} , z = 0 , \omega\left( k_{x} , k_{y} , k_{z} \right) \right)e^{ik_{x}x}e^{ik_{y}y}e^{ik_{z}z}ck_{z}/\sqrt{k_{x}^{2}+k_{y}^{2}+k_{z}^{2}}dk_{x}dk_{y}dk_{z}.$ (13)

Although Eq. 12 is in the form of a 3-D inverse Fourier transform, the non-uniform grid of $k_{z}$ may cause numerical artifacts. A more accurate solution is using NUFFT to upgrade Eq.12 as

$S\left( x , y , z \right)=\iiint P\left( k_{x} , k_{y} , z = 0 , \omega\left( k_{x} , k_{y} , k_{z} \right) \right)e^{ik_{x}x}e^{ik_{y}y}e^{ik_{z}z}ck_{z}/\sqrt{k_{x}^{2}+k_{y}^{2}+k_{z}^{2}}\Phi D_{p}dk_{x}dk_{y}dk_{z},$ (14)

where $\Phi$ is a sinc interpolation basis and $D_{p}$ is a down-sampling function. The main drawback of NUFFT is that it was only applied to homogenous media. For heterogeneous media, the wave propagation also could be easily expressed in the wavenumber domain. Assuming an *L*-th layered media as shown in Supplementary Figure 31a, the backpropagation in Eq.7 is rewritten as

$P\left( k_{x} , k_{y} , z_{s} , \omega\right)=P\left( k_{x} , k_{y} , \omega, z = 0 \right)\prod_{l=1}^{L-1} e^{ik_{l}d_{l}}e^{ik_{L}\left( z_{s} - z_{L} \right)},$ (15)

where $k_{q}$, $k_{L}$ and $z_{L}$ are defined as

$$k_{l}=\sqrt{{\omega^{2}}/{c_{l}^{2}}-k_{x}^{2}-k_{y}^{2}},$$

$$k_{L}=\sqrt{{\omega^{2}}/{c_{L}^{2}}-k_{x}^{2}-k_{y}^{2}},$$

$$z_{L}=\sum_{l=1}^{L-1} d_{l}.$$

It can be seen that Eq. 14 is similar to Eq. 7 but with one more phase term of $\prod_{l=1}^{L-1} e^{ik_{l}d_{l}}$. Therefore, a direct solution is to use phase shift (PS) migration to extrapolate the wavefield to depth $z_{L}$ and then implement NUFFT to achieve the source term in the *L*-th layer as

$S\left( x , y , z \right)=NUFFT\left\{ P \left( k_{x} , k_{y} , \omega, z = 0 \right) \prod_{l=1}^{L-1} e^{ik_{l}d_{l}} \right\}.$ (16)

The whole flow-chart of PS-NUFFT is shown in Supplementary Figure 31b. Noted that, for an *L*-th layered media, Eq.14 should be implemented *L* times for imaging the whole region (from layer 1 to layer *L*). The current PS-NUFFT can only apply to OA imaging but not to ultrasound imaging due to the precondition of one-way propagation.


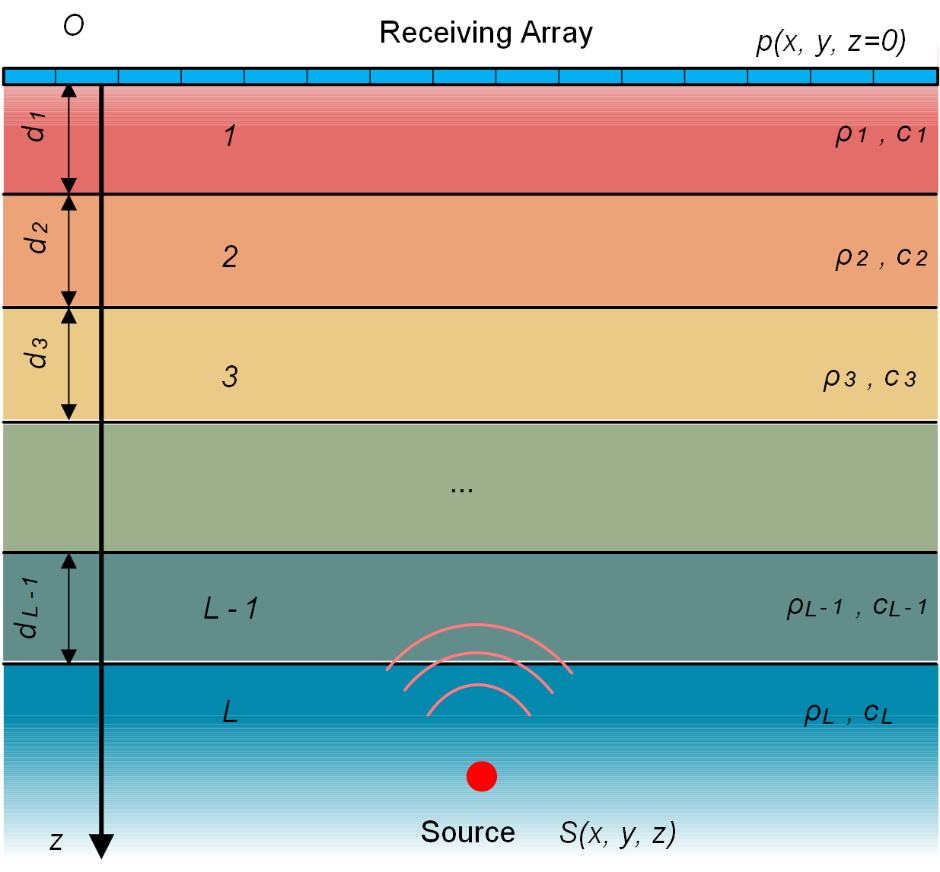


**Supplementary Figure 31a. The *L*-th layered media with an acoustic source at depth *z*.**


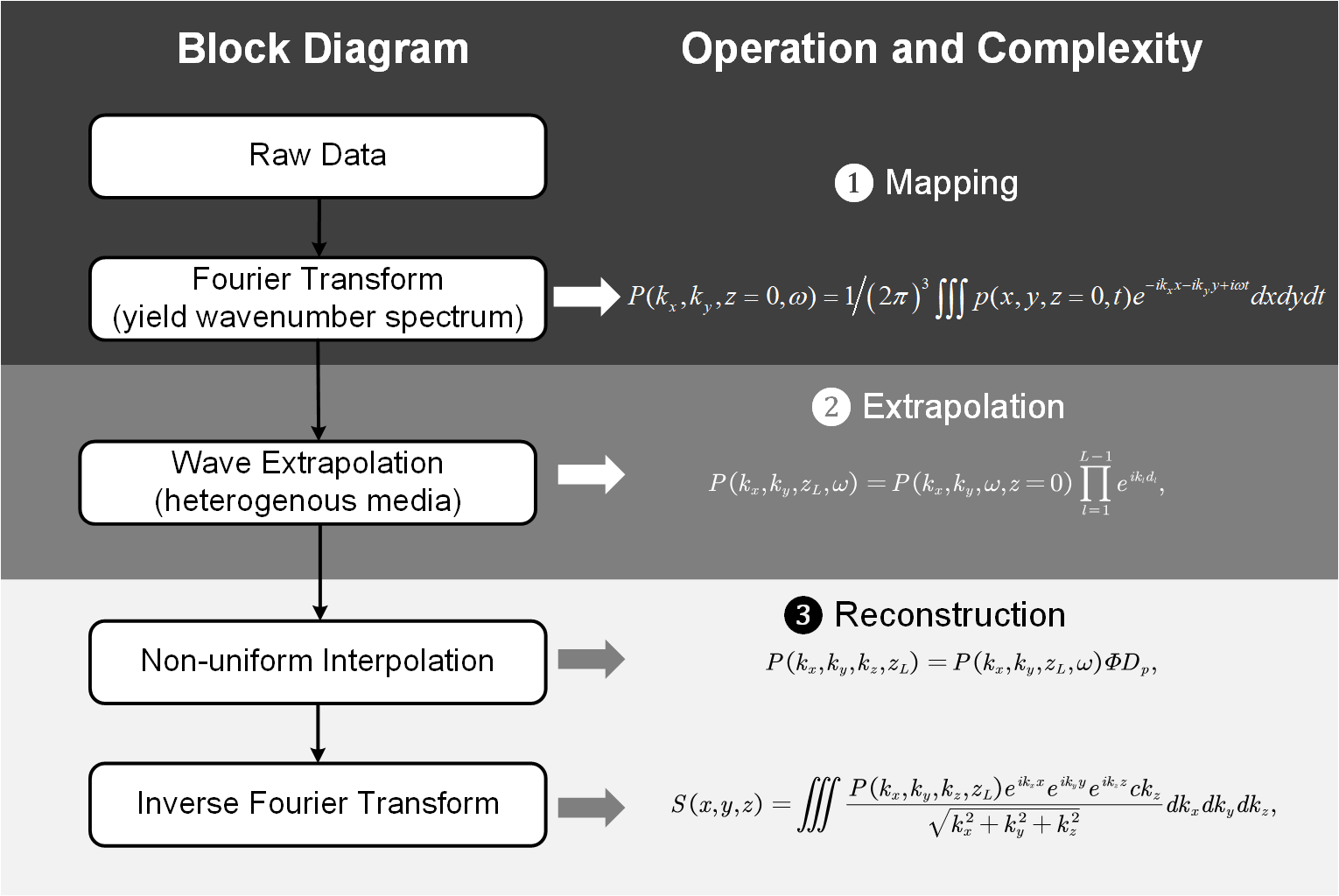


**Supplementary Figure 31b. The flow-chart of PS-NUFFT.**

**Supplementary Note 15. Simulation for 3D imaging algorithm verification.**

The simulation is based on the phantom of the blood vessel, and its data are generated using *K-wave 1.2.1 Matlab 2017b* [35]. The media is set as a two-layered media, as shown in Supplementary Figure 32. Its upper layer is defined as the dermis (skin) with a sound speed of 1730 m/s, a density of 1150 kg/m^3^ and a thickness of 2.2 mm, while the bottom layer is the hypodermis layer filled with fat having a sound speed of 1450 m/s, a density of 950 kg/m^3^and a thickness of 3.2 mm. The blood vessel is buried in a depth of 5 mm. All the sensor elements are aligned at a depth of 0 mm with a pitch of 0.1 mm and a total range of 25.6 mm. The ultrasound waves are recorded from 0 µs to 7 µs with a sampling frequency of 60 MHz (total 420 sampling points).


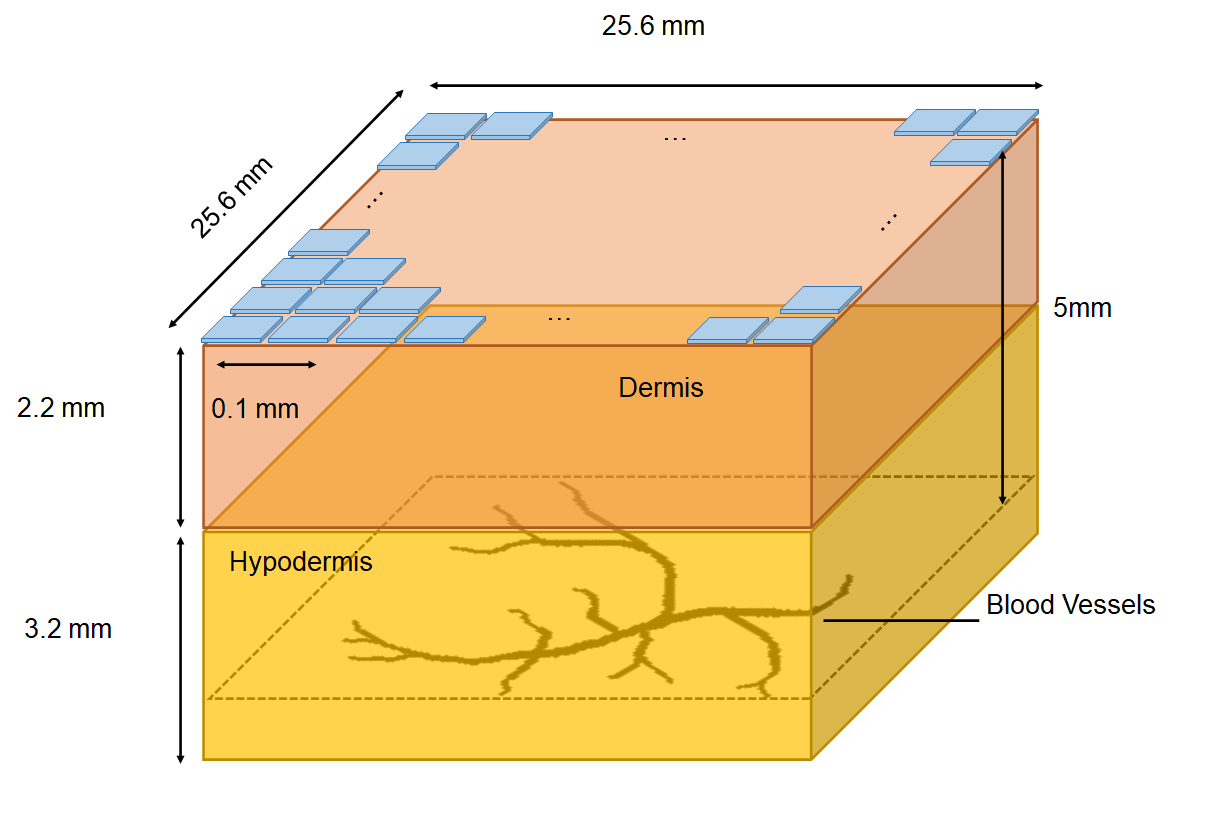


**Supplementary Figure 32. Simulation Phantom.** The acoustic sources (blood vessels) are buried in a two-layered media at a depth 5 mm. The top layer of media is the dermis with a sound speed of 1730 m/s, a density of 1150 kg/m^3^ and a thickness of 2.2 mm. The bottom layer is hypodermis with a sound speed of 1450 m/s, a density of 950 kg/m^3^ and a thickness of 3.2 mm.

**Supplementary Note 16. Reconstruction performance of PS-NUFFT vs. DAS with the actual sensor parameters.**

The simulations with the actual sensor parameters were done on single-layer, two-layer, and three-layer media configurations to demonstrate the generalizability of the proposed PS-NUFFT approach (Supplementary Figure 33). All the simulation data are generated from the k-wave toolbox in Matlab 2022b. The phantoms are configured as single-layer (sound speed $c=1540m/s$), two-layers ($c=1750m/s$ and $c=1450m/s$) and three-layers media ($c=1450m/s$, $c=1540m/s$ and $c=1450m/s$) in these three simulations, as shown in Supplementary Figure 33(a). Three targets of “L”, “Z” and “M” letter shapes are placed at depth of 5 mm in the media as optoacoustic sources. All the optoacoustic waves are recorded by the 6*6 acoustic nodes with each node size of 1.6mm*1.6mm. The sampling frequency is 100 MHz along with a sampling length of 20μs.

All the results are double interpolated for better visualization. The raw images are shown in Supplementary Figure 33(b) where the target letters are hardly distinguished. The images reconstructed with classical DAS are presented in Supplementary Figure 33(c). Due to the unresolved effects of small aperture and heterogenous media, classical DAS failed to reconstruct high-quality images. In comparison with DAS, our methods can effectively reconstruct the target letters’ morphology as shown in Supplementary Figure 33(d). With the actual sensor specifications, the improvement in image quality is once again remarkable.


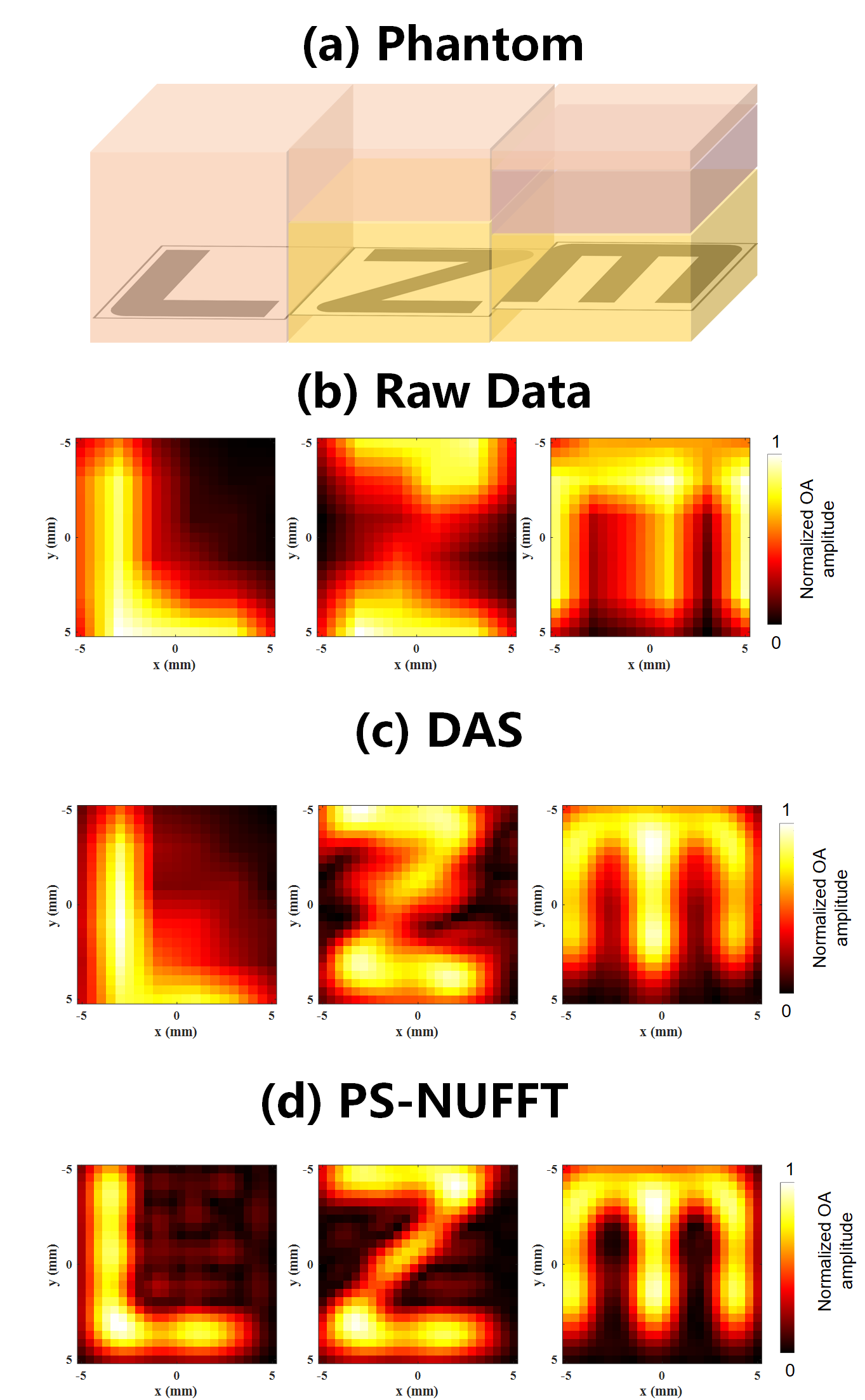


**Supplementary Figure 33. Reconstruction simulations with actual sensor parameters.** **(a)** The layered simulation settings. **(b)** The raw data images. **(c)** The DAS-reconstructed images. **(d)** The PS-NUFFT-reconstructed images.

**Supplementary Note 17. Reconstruction performance of PS-NUFFT under bending conditions.**

To validate the reconstruction performance of PS-NUFFT for OBS under bending conditions, three sets of simulations are provided. The simulation setup is the same as supplementary Note 16, except the sensor is under different bending conditions (R = 100 mm, R = 50mm and R = 20 mm). The image results are shown in Supplementary Figure 34. It can be seen that the bending of the OBS will lead to different degrees of distortion of the imaging results (Supplementary Figure 34(b), (d) and (f)). The image quality further deteriorates with the decreasing bend radius. The main reason is the acoustic nodes are not at a plane. Fortunately, these distortions could be avoided by incorporating the pre-migration technique [36] into PS-NUFFT. After compensating for the deviation from the positions of acoustic nodes, the image results are restored as shown in Supplementary Figure 34(c), (e) and (g).


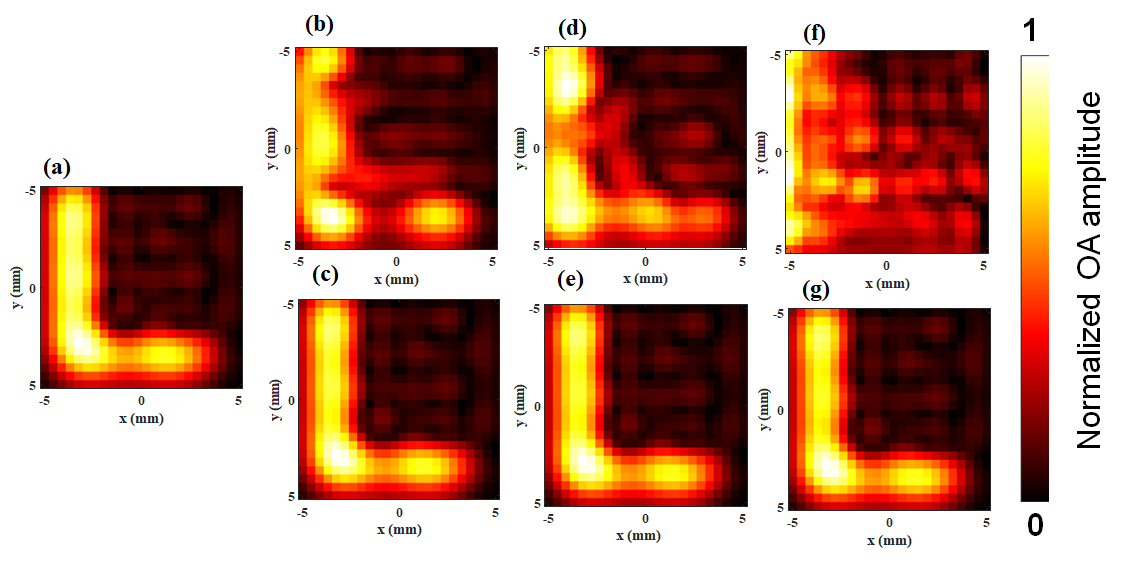


**Supplementary Figure 34. Simulation of reconstructed images before and after compensation. (a)** Unbent condition. **(b)** Image at 100mm bending radius before compensation. **(c)** Image at 100 mm bending radius after compensation. **(d)** Image at 50 mm bending radius before compensation. **(e)** Image at 50 mm bending radius after compensation. **(f)** Image at 20 mm bending radius before compensation. **(g)** Image at 20 mm bending radius after compensation.

**Supplementary Note 18. Image depth of the OBS.**

Different from classical optical imaging modality, optoacoustic imaging is able to image blood information in deep [37]. To verify the imaging depth of our proposed optoacoustic blood stethoscope, both ex-vivo and in-vivo experiments are conducted. The light source system and data acquisition system are all following the description in the method section. In the ex-vivo experiments, black tapes as targets are buried under different pork slice phantoms (indicated with enclosed dotted rectangles. According to the recorded results, it can be seen that our optoacoustic stethoscope can easily detect the targets at 10 mm depth.


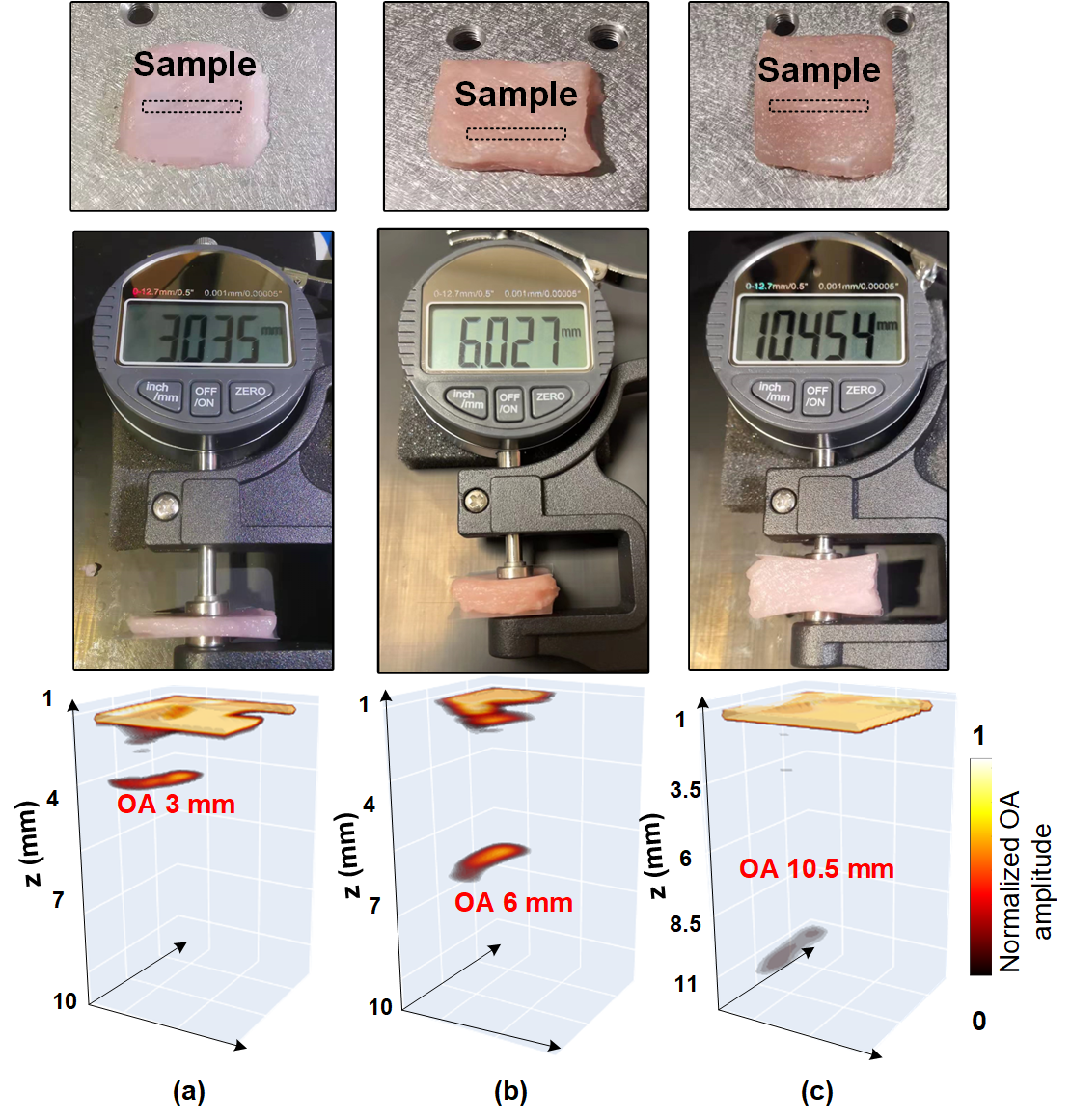


**Supplementary Figure 35. Ex-vivo deep penetration experiment.** (a) 3 mm penetration experiment. (b) 6 mm penetration experiment. (c) 10 mm penetration experiment.

Meanwhile, measurements of imaging resolution and SNR at different depths ranging from 1.5mm to 13 mm are conducted with a similar experimental setup and protocol but with line targets at various depths. Supplementary Figure 36 compares the lateral resolution and SNR from the results before and after reconstructions, where the resolutions are determined by Full Width at Half Maximum value (FWHM). It should be noted that due to acoustic diffraction, the image resolution of raw data decreases as imaging depth increases. In contrast, the proposed reconstruction method can retain imaging resolution at various depths, given that the SNR is sufficient.


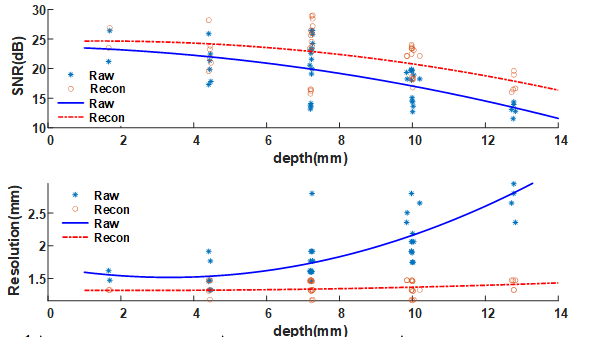


**Supplementary Figure 36. The SNR (a) and resolution (b) of line targets at various depths.** (Raw: Raw data; Recon: Reconstructed value; dots and lines stand for the measured and curve-fitted data)

Measurement of blood vessels in the forearm was conducted to evaluate the in vivo penetration ability; the result in Supplementary Figure 37 shows that our sensor can still detect considerable PA signals from deep blood arteries (even at a depth of around 6 mm).


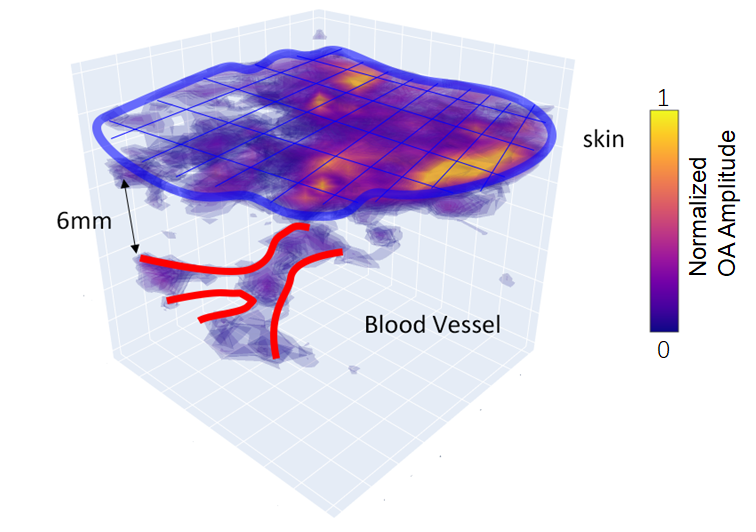


**Supplementary Figure 37. The deep blood vessels imaging on the forearm (~ 6 mm) with the proposed OBS.**

In practice, it should make clear that although the OBS can probe relatively deep blood arteries, the images have limited pixel resolution and minor SNR, making the diagnosis rely heavily on professional judgment to avoid any potential misinterpretations. However, this result does not establish the maximum in-vivo detection depth since it depends on several factors, including skin tone, probing laser wavelength, absorption spectra of the contrast agent, etc. Generally, combining the NIR-II light source with high penetrability and highly absorptive exogenous contrast agents/drugs will greatly extend the in-vivo working distance to several centimeters [11].

**Supplementary Note 19.** **The scope of optoacoustic ‘drugs’.**

Photoacoustic imaging is grounded on the optical absorption properties of objects; hence, not all drugs could be detected. More specifically, they can be classified into the following two categories: 1. the drugs modified or combined (tagged) with optoacoustic contrast. For instance, drugs can be combined with RhB tags for tracing purposes, 2. the drugs capable of self-generating strong optoacoustic signals.

Nowadays, drugs have been modified to carry contrast agents to introduce the visualizing capability for diagnosing purposes among various medical imaging techniques, such as positron emission tomography (PET), X-ray computed tomography (CT), magnetic resonance imaging (MRI), ultrasound (US) imaging, and optical imaging. These image-guided drug delivery systems use medical imaging techniques to optimize therapeutic efficacy by maximizing accumulation in the diseased tissue while minimizing drug toxicity in the surrounding healthy tissue. Optical imaging techniques (including fluorescence imaging) are widespread for drug delivery monitoring, and they are advantageous in small animal studies because they can provide molecular functional information in biological tissues.

Indeed, a strong optical absorber at the targeted wavelengths is a prerequisite for optoacoustic imaging. Although some drugs themselves may not absorb much light, they can be loaded with strong absorbing nanoagents for image-guided drug delivery. Following this idea, researchers have modified the drug molecules by incorporating various chromophores to enhance their optical absorbances, such as resveratrol (drug) with ion oxide (OA chromophores) for stem cell therapy [38], camptothecin (drug) with cyanine-based photocage (OA chromophores) for photolysis therapy [39] and DC101 (drug) with intrinsic hemoglobin (OA chromophores) for anti-angiogenic therapy [40]. Especially for tumor treatment, the concentration of drugs bonded with optoacoustic contrast [41-43] can be facilely monitored and controlled for tumor eradication. In this study, we use RhB as a model chromophore to demonstrate the device’s capability of monitoring such drug labelling probes [44-47].

The second type of 'drugs' is the absorbing ones that can also act as optoacoustic agents. For example, some photothermal agents [15, 16] exhibiting strong optoacoustic response have been applied in optoacoustic imaging coupled with photothermal therapy (PTT) owing to their high absorption coefficient, good photostability, and suitable biocompatibility.

**Supplementary Note 20. The sensitivity of RHB detection**.

Drugs like Rhodamine B (RHB) can be detected by our OBS for monitoring its decay line. In this note, phantom experiment and Monte Carlo simulation are presented to estimate the sensitivity of RHB monitoring. Solutions with different RHB concentrations are perfused into quartz cuvettes as the phantom for testing. A 532 nm laser is employed as the light source to excite the optoacoustic signals. Since quartz cuvette is highly optically transparent at 532 nm (broadband transmission> 95%), it can be reasonably considered as a non-attenuated case. According to Supplementary Figure 38(a), the baseline detectable concentration sensitivity of RHB by our optoacoustic blood stethoscope is within 30 *μg/mL* – 2000 *μg/mL*. For lower concentrations, the results become indistinguishable. However, for the in-vivo experiment, we should not neglect optical attenuation and scatter in the tissue, where such an energy loss can be simulated by the Monte Carlo method. In Supplementary Figure 38(b) and (c), the Monte Carlo result shows that the laser energy loss is about -15 *dB/mm*. Combining both the phantom and simulation results, the in-vivo minimum detectable concentration will be further reduced to about $30\times5.6^{\alpha}\mu g/mL$ at a depth of *α* mm, under a 532 nm laser sources.


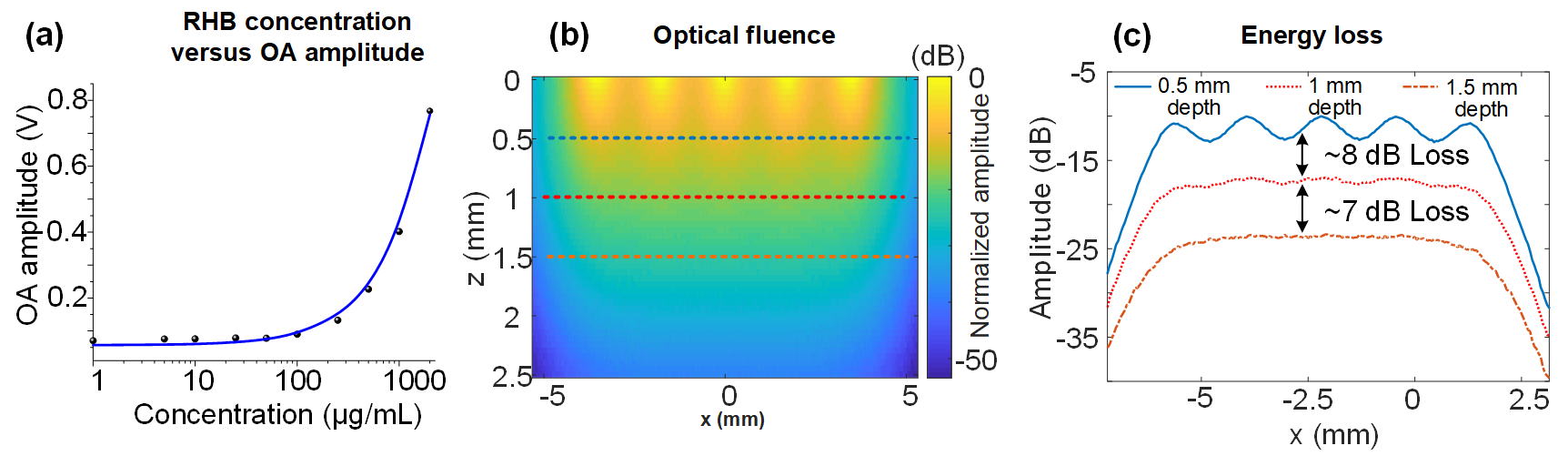


**Supplementary Figure 38**. **(a)** Correlation between RHB concentration and OA amplitude. **(b)** Optical fluence in tissue. **(c)** Optical energy loss at different depths.

**Supplementary Note 21. Fluorescence test of the decay rate of Rhodamine B.**

Rhodamine B is able to fluoresce and can be detected by fluorometers. The fluorescence test was in vitro testing with a monochromator microplate reader (Infinile M200, TECAN); hence, the mice blood should be invasively sampled.

*Sample Preparation:* Before the Rhodamine B was injected into the mouse, we took 5 µL blood from the mouse tail twice as the baseline. After the Rhodamine B was injected, the mouse was taken 5 µL of blood from its tail every 5 mins. The whole process continued for 2 hours, and 24 blood samples were collected. Every blood sample was mixed with a 5 µL blood anticoagulant after drawing out from the tail and then diluted with 190 µL water to provide sufficient liquid volumes for fluorescence test (Supplementary Figure 39a).


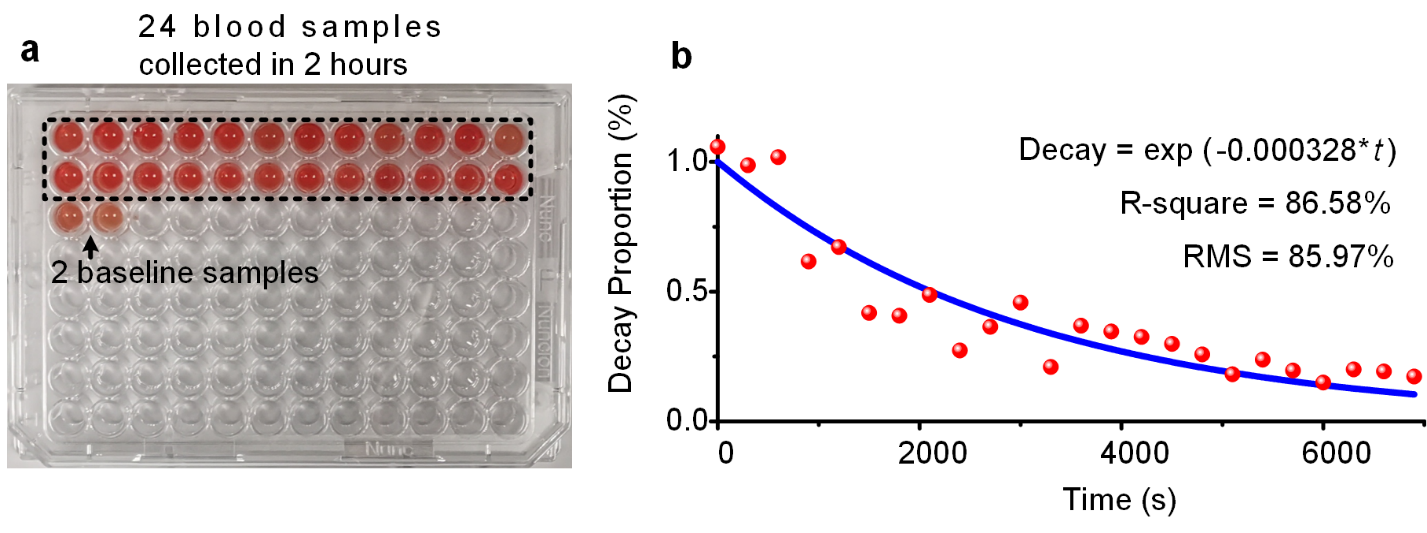


**Supplementary Figure 39. Fluorescence tests. a,** The blood samples**. b,** The decay trendline of Rhodamine B estimated by fluorescence tests.

*Fluorescence Test:* The excitation light wavelength was set as 560 nm with a bandwidth of 9 nm, and the tested emission wavelength was set as 590 nm with a bandwidth of 20 nm. The number of flashes was 25 with an integration time of 20 µs. This test was under an ambient temperature of 23.2 °C. The total decay rate of Rhodamine B was about -3.28×10^-4^ per second (Supplementary Figure 39b). In comparison, the decay rate of Rhodamine B measured by fluorescence test at 3600 s – 5700 s was about -3.22×10^-4^ per second, which was closed to our OBS’s monitored result of -3.61×10^-4^ per second.

**Supplementary Note 22.**  **The impact of skin tones on the measurements**.

The skin PA amplitudes variation measured at the dorsal forearm was observed across different skin tones, as quantified and compared in Supplementary Table 6. This factor will undoubtedly affect the light penetration and herein the absolute blood PA signal reading. However, rather than absolute PA amplitude, our time evolution analysis is based on the ‘changes in percentage’. Such measurements are self-referenced (i.e., independent of absolute baseline), assuring more reproducible and comparable results across individuals and measurement settings, i.e. the different skin tones will not affect the percentage readings. More specifically, this internal calibration mitigates the discrepancies in absolute amplitude variation relative to the baseline value, caused by skin tones and a list of other complex in-vivo factors.

**Supplementary Table 6. The effects of skin tone on the skin OA amplitudes** **at the dorsal forearm (n = 3).**

|  | Subject 1  (Fitz. Type 4) | Subject 2  (Fitz. Type 3) | Subject 3  (Fitz. Type 2) |
| --- | --- | --- | --- |
| Normalized skin PA signal at dorsal forearm | 1± 0.15 | 0.95± 0.04 | 0.82± 0.03 |

**Supplementary Note 23**. **Skin bending and motion artifacts**

Before attaching the sensors to the dorsal hand, the skin surface curvature is measured with a magnetic marking pen (Ascension 3D Guidance drive BAY) for one-time calibration, therefore the skin surface information could be registered into coordinates. As depicted in Supplementary Figure 40, stereoviews can be created from measured point clouds; hence, the element positions in the ROI can be inferred. The skin surface profile can also be captured using any other stereo device, such as the LiDAR module on a smartphone or a stereo camera. With the development of microelectronics, it is promising that future LiDAR chips can be downsized and integrated into a flexible substrate to serve this function. When the OBS is attached to the limbs distant from joints and muscle groups, it is reasonable to assume that the surface is relatively smooth; as the skin bending around the forearm, dorsal hand, and thigh are all safely above 20 mm, which is the minimum radius considered in this work. Since OBS is not stretchable, the relative positions between acoustic elements are relatively immobilized.


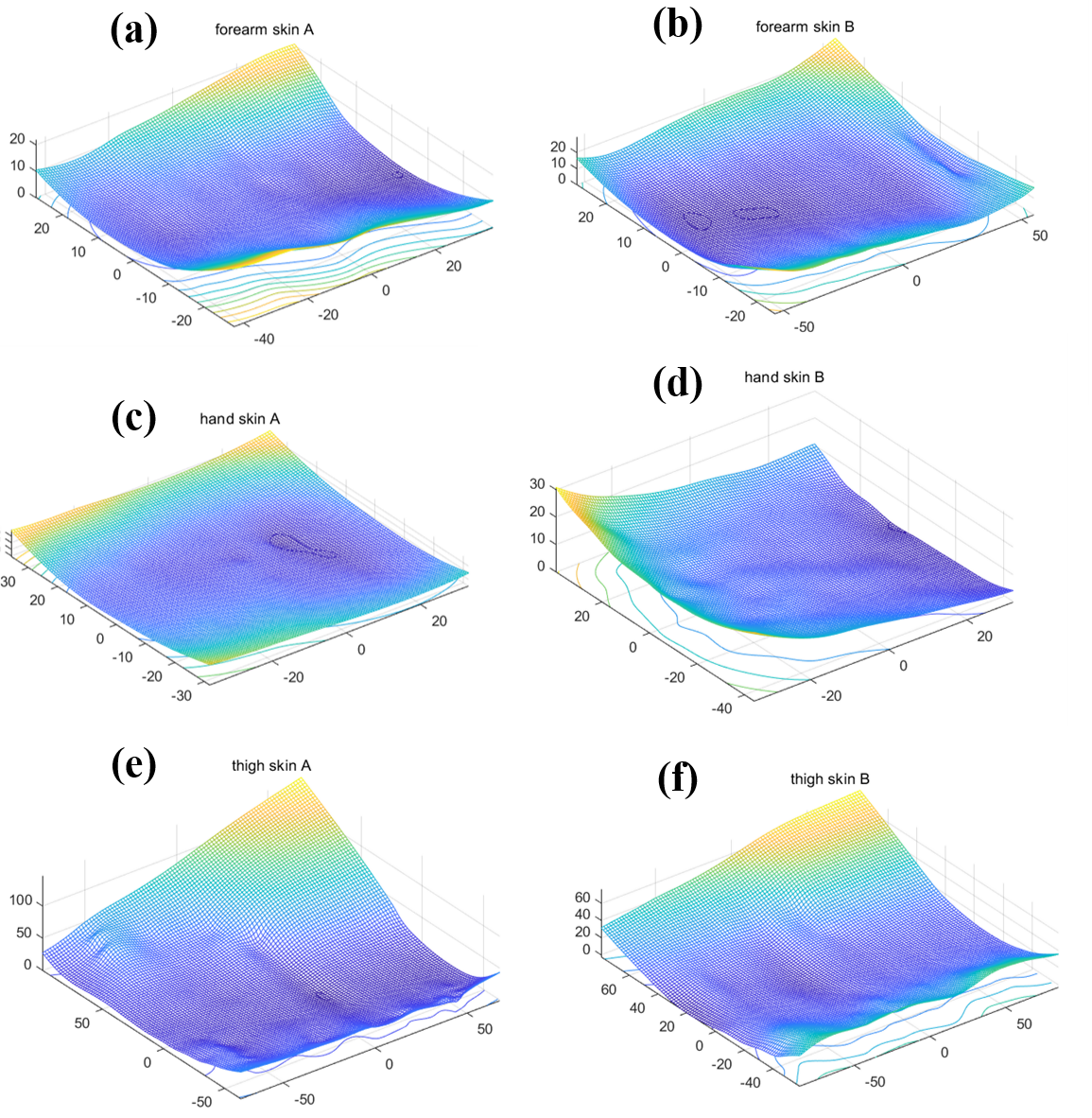


**Supplementary Figure 40. The stereoview of the forearm** **(a,b**), dorsal hand **(c,d)** and thigh **(e,f)** skin profiles.

In the context of biomedical imaging, two types of motion artifacts of particular interest must be addressed. The first type is subject to involuntary motions such as respiration and heartbeat. The respiratory gating approach can minimize artifacts from breathing and heartbeat [48]. In our trials, the sensor was measured at a considerable distance from the chest; hence, the involuntary movement did not cause any artifacts.

The other type is induced by active movements, such as the skin bending during the course of measurement, which may change the blood vessels' locations relative to the skin and hence the signal amplitude. The proposed sensor belongs to the PA tomography modality, which can concurrently acquire the waveforms from all channels across the whole ROI in a single nanosecond laser shot period. On the one hand, no progressive scanning-related visual distortions such as the jello effect, which are common in PA microscopy and other rolling shutter imaging modalities, were observed with our tomographic sensor. On the other hand, imaging with a single laser pulse prevents blurring caused by motion averaging, analogous to global shutter imaging with ultrashort-time exposure. Even for applications requiring multiple-frame average imaging to improve SNR, techniques such as motion error compensation have been explored to address this issue [49-51].

Last but not least, a proper adhesion technique can be applied to further minimize the motion artifacts. The sensor is attached to the skin using medical double-sided tape which replaces the encapsulation around the optical and acoustic node region. While the PVDF membrane is flexible, it isn't sufficiently conformable, hence air gap can present due to the geometric mismatch between the film and the skin. In our design, this is partially improved by introducing a thin soft PDMS encapsulation layer that can deform to couple this mismatch, thereby enhancing contact. For short-term applications, a coupling gel beneath the probe is employed to mitigate the presence of air gaps. However, traditional ultrasound gel may dry out over time, which could potentially attenuate the signal amplitude. As suggested in [52], uncured silicone is a viable alternative for extended use due to its maintained coupling efficiency over time. It is challenging that intense movements can still introduce air gaps, compromise ultrasonic coupling, and potentially cause the sensor patch to detach, all of which can lead to signal loss. Recent advancements in the field have introduced a bioadhesive hydrogel-elastomer [9]. This novel material provides both durable ultrasonic coupling and strong adhesion, effectively addressing the aforementioned issues. Integrating this material into our probe design is expected to effectively eliminate potential air gaps, thereby ensuring robust long-term performance, even under conditions of vigorous motion.

Our current design is intended for smooth skin areas with minimal curvature, making it unsuitable for highly curved regions like human joints. However, when adhered correctly, no air-bubble-induced signal loss was observed during measurements, indicating that the interface can adjust to subtle skin curvature variations due to translation movement, slight rotational motion, and involuntary motions such as respiration and heartbeat.

Nonetheless, movements causing dynamic skin bending would affect the sensor element's deformation and the positional relationship of blood vessels relative to the skin, thus influencing the optoacoustic signals of blood vessels. This type of motion artifact originates from the dynamic sensor distribution, which can be mitigated by incorporating an additional strain sensor and algorithm, as discussed in Supplementary Note 17.

**Supplementary Note 24. Medical ultrasound image processing.**

The processing of medical ultrasound images mainly includes six steps: window selection, binarization, morphological operations, edge outline and filling. In Supplementary Figure 41a, a window of 80*140 pixels was used to select the artery image out from the medical ultrasound data. The blood vessels are usually presented as shadows in ultrasound images. Then, the selected image was binarized, as shown in Supplementary Figure 41b. To remove the small gaps and discontinuities, two morphological open and one close operation were applied to the binary image as plotted in Supplementary Figure 41c. Subsequently, an edge operation was employed to extract the artery profile. The arterial cross-sectional area could be conveniently estimated with the filled profile as depicted in Supplementary Figure 41d.


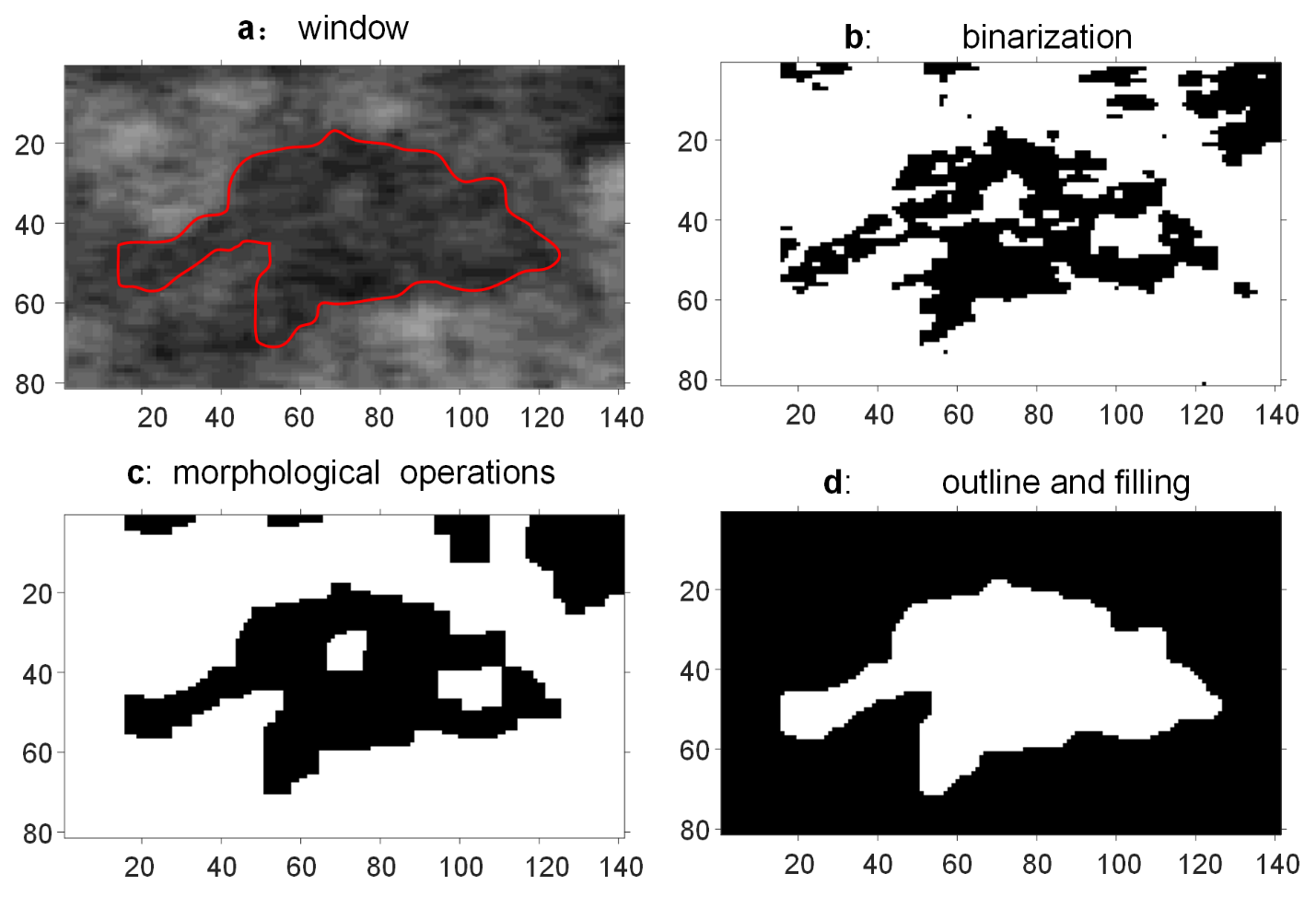


**Supplementary Figure 41. The image processing of ultrasound image. a,** Selection ROI with a specific window. **b,** Binarize ultrasound image. **c,** Removing small discontinuities with morphological operations. **d,** Removing large gaps by filling the outlines.

**Supplementary References**

[1] L. Brekhovskikh, *Waves in layered media*. Elsevier, 2012.

[2] W. S. Gan, *Acoustical imaging: techniques and applications for engineers*. John Wiley & Sons, 2012.

[3] C. M. Lochner, Y. Khan, A. Pierre, and A. C. Arias, "All-organic optoelectronic sensor for pulse oximetry," *Nature communications,* vol. 5, no. 1, pp. 1-7, 2014.

[4] X. Xing and M. Sun, "Optical blood pressure estimation with photoplethysmography and FFT-based neural networks," *Biomedical optics express,* vol. 7, no. 8, pp. 3007-3020, 2016.

[5] H. Teymourian, A. Barfidokht, and J. Wang, "Electrochemical glucose sensors in diabetes management: An updated review (2010–2020)," *Chemical Society Reviews,* vol. 49, no. 21, pp. 7671-7709, 2020.

[6] L. Klous, C. De Ruiter, S. Scherrer, N. Gerrett, and H. Daanen, "The (in) dependency of blood and sweat sodium, chloride, potassium, ammonia, lactate and glucose concentrations during submaximal exercise," *European journal of applied physiology,* vol. 121, no. 3, pp. 803-816, 2021.

[7] W. Gao *et al.*, "Fully integrated wearable sensor arrays for multiplexed in situ perspiration analysis," *Nature,* vol. 529, no. 7587, pp. 509-514, Jan 28 2016, doi: 10.1038/nature16521.

[8] K. Meng *et al.*, "Flexible weaving constructed self‐powered pressure sensor enabling continuous diagnosis of cardiovascular disease and measurement of cuffless blood pressure," *Advanced Functional Materials,* vol. 29, no. 5, p. 1806388, 2019.

[9] C. Wang *et al.*, "Bioadhesive ultrasound for long-term continuous imaging of diverse organs," *Science,* vol. 377, no. 6605, pp. 517-523, 2022.

[10] C. Wang *et al.*, "Monitoring of the central blood pressure waveform via a conformal ultrasonic device," *Nature biomedical engineering,* vol. 2, no. 9, pp. 687-695, 2018.

[11] L. V. Wang and J. Yao, "A practical guide to photoacoustic tomography in the life sciences," *Nat Methods,* vol. 13, no. 8, pp. 627-38, Jul 28 2016, doi: 10.1038/nmeth.3925.

[12] I. Ivankovic, E. Merčep, C.-G. Schmedt, X. L. Deán-Ben, and D. Razansky, "Real-time volumetric assessment of the human carotid artery: handheld multispectral optoacoustic tomography," *Radiology,* vol. 291, no. 1, pp. 45-50, 2019.

[13] X. L. Deán-Ben and D. Razansky, "Functional optoacoustic human angiography with handheld video rate three dimensional scanner," *Photoacoustics,* vol. 1, no. 3-4, pp. 68-73, 2013.

[14] M. S. Losch, F. Kardux, J. Dankelman, and B. H. Hendriks, "Steering light in fiber-optic medical devices: a patent review," *Expert Review of Medical Devices,* vol. 19, no. 3, pp. 259-271, 2022.

[15] A. K. Bindra *et al.*, "Self-assembled semiconducting polymer based hybrid nanoagents for synergistic tumor treatment," *Biomaterials,* vol. 279, p. 121188, 2021.

[16] L. Zhao, X. Zhang, X. Wang, X. Guan, W. Zhang, and J. Ma, "Recent advances in selective photothermal therapy of tumor," *J Nanobiotechnology,* vol. 19, no. 1, p. 335, Oct 24 2021, doi: 10.1186/s12951-021-01080-3.

[17] M. R. Querry, *Optical constants of minerals and other materials from the millimeter to the ultraviolet*. Chemical Research, Development & Engineering Center, US Army Armament …, 1987.

[18] J. J. Niederhauser, M. Jaeger, M. Hejazi, H. Keppner, and M. Frenz, "Transparent ITO coated PVDF transducer for optoacoustic depth profiling," *Optics Communications,* vol. 253, no. 4-6, pp. 401-406, 2005, doi: 10.1016/j.optcom.2005.05.005.

[19] Z. Xiao, O. Adelegan, F. Y. Yamaner, and O. Oralkan, "CMUTs on glass with ITO bottom electrodes for improved transparency," presented at the 2016 IEEE International Ultrasonics Symposium (IUS), 2016.

[20] C. Fang, H. Hu, and J. Zou, "A Focused Optically Transparent PVDF Transducer for Photoacoustic Microscopy," *IEEE Sensors Journal,* 2019.

[21] J. J. Niederhauser, M. Jaeger, M. Hejazi, H. Keppner, and M. Frenz, "Transparent ITO coated PVDF transducer for optoacoustic depth profiling," *Optics Communications,* vol. 253, no. 4, pp. 401-406, 2005/09/15/ 2005, doi: <https://doi.org/10.1016/j.optcom.2005.05.005>.

[22] X. Zhang, O. Adelegan, F. Y. Yamaner, and O. Oralkan, "CMUTs on glass with ITO bottom electrodes for improved transparency," in *2016 IEEE International Ultrasonics Symposium (IUS)*, 2016: IEEE, pp. 1-4.

[23] X. Zhang, X. Wu, O. J. Adelegan, F. Y. Yamaner, and Ö. Oralkan, "Backward-mode photoacoustic imaging using illumination through a CMUT with improved transparency," *IEEE transactions on ultrasonics, ferroelectrics, and frequency control,* vol. 65, no. 1, pp. 85-94, 2017.

[24] Z. Li, A. K. Ilkhechi, and R. Zemp, "Transparent capacitive micromachined ultrasonic transducers (CMUTs) for photoacoustic applications," *Optics express,* vol. 27, no. 9, pp. 13204-13218, 2019.

[25] A. Dangi, S. Agrawal, and S.-R. Kothapalli, "Lithium niobate-based transparent ultrasound transducers for photoacoustic imaging," *Optics letters,* vol. 44, no. 21, pp. 5326-5329, 2019.

[26] Q. Fang and D. A. Boas, "Monte Carlo simulation of photon migration in 3D turbid media accelerated by graphics processing units," *Optics express,* vol. 17, no. 22, pp. 20178-20190, 2009.

[27] S. L. Jacques, "Coupling 3D Monte Carlo light transport in optically heterogeneous tissues to photoacoustic signal generation," *Photoacoustics,* vol. 2, no. 4, pp. 137-142, 2014.

[28] M. Xu and L. V. Wang, "Photoacoustic imaging in biomedicine," *Review of scientific instruments,* vol. 77, no. 4, p. 041101, 2006.

[29] L. F. Brown and J. L. Mason, "Disposable PVDF ultrasonic transducers for nondestructive testing applications," *IEEE Transactions on Ultrasonics, Ferroelectrics, and Frequency Control,* vol. 43, no. 4, pp. 560-568, 1996, doi: 10.1109/58.503716.

[30] L. F. Brown, "Design considerations for piezoelectric polymer ultrasound transducers," *IEEE transactions on ultrasonics, ferroelectrics, and frequency control,* vol. 47, no. 6, pp. 1377-1396, 2000.

[31] S.-C. Wooh and Y. Shi, "Three-dimensional beam directivity of phase-steered ultrasound," *The Journal of the Acoustical Society of America,* vol. 105, no. 6, pp. 3275-3282, 1999.

[32] H. Jin, R. Zhang, S. Liu, and Y. Zheng, "Fast and High-Resolution Three-Dimensional Hybrid-Domain Photoacoustic Imaging Incorporating Analytical-Focused Transducer Beam Amplitude," *IEEE Transactions on Medical Imaging,* vol. 38, no. 12, pp. 2926-2936, 2019, doi: 10.1109/TMI.2019.2917688.

[33] H. Jin, R. Zhang, S. Liu, and Y. Zheng, "Rapid Three-Dimensional Photoacoustic Imaging Reconstruction for Irregularly Layered Heterogeneous Media," *IEEE Transactions on Medical Imaging,* vol. 39, no. 4, pp. 1041-1050, 2020, doi: 10.1109/TMI.2019.2940757.

[34] H. Jin, S. Liu, R. Zhang, S. Liu, and Y. Zheng, "Frequency Domain Based Virtual Detector for Heterogeneous Media in Photoacoustic Imaging," *IEEE Transactions on Computational Imaging,* vol. 6, pp. 569-578, 2020, doi: 10.1109/TCI.2020.2964240.

[35] B. E. Treeby and B. T. Cox, "k-Wave: MATLAB toolbox for the simulation and reconstruction of photoacoustic wave fields," *J Biomed Opt,* vol. 15, no. 2, p. 021314, Mar-Apr 2010, doi: 10.1117/1.3360308.

[36] H. Jin *et al.*, "Pre-migration: A General Extension for Photoacoustic Imaging Reconstruction," *IEEE Transactions on Computational Imaging,* vol. 6, pp. 1097-1105, 2020, doi: 10.1109/tci.2020.3005479.

[37] L. H. V. Wang and S. Hu, "Photoacoustic Tomography: In Vivo Imaging from Organelles to Organs," *Science,* vol. 335, no. 6075, pp. 1458-1462, Mar 2012, doi: 10.1126/science.1216210.

[38] I. M. Adjei *et al.*, "Multifunctional nanoparticles for intracellular drug delivery and photoacoustic imaging of mesenchymal stem cells," *Drug delivery and translational research,* vol. 9, no. 3, pp. 652-666, 2019.

[39] Y. Zhang *et al.*, "Harnessing Hypoxia‐Dependent Cyanine Photocages for In Vivo Precision Drug Release," *Angewandte Chemie,* vol. 133, no. 17, pp. 9639-9647, 2021.

[40] H.-C. Zhou *et al.*, "Optical-resolution photoacoustic microscopy for monitoring vascular normalization during anti-angiogenic therapy," *Photoacoustics,* vol. 15, p. 100143, 2019.

[41] Q. Wang *et al.*, "All‐in‐one phototheranostics: single laser triggers NIR‐II fluorescence/photoacoustic imaging guided photothermal/photodynamic/chemo combination therapy," *Advanced Functional Materials,* vol. 29, no. 31, p. 1901480, 2019.

[42] X. Yao *et al.*, "Multifunctional nanoplatform for photoacoustic imaging-guided combined therapy enhanced by CO induced ferroptosis," *Biomaterials,* vol. 197, pp. 268-283, 2019.

[43] Y. He *et al.*, "Multifunctional polypyrrole‐coated mesoporous TiO2 nanocomposites for photothermal, sonodynamic, and chemotherapeutic treatments and dual‐modal ultrasound/photoacoustic imaging of tumors," *Advanced healthcare materials,* vol. 8, no. 9, p. 1801254, 2019.

[44] E. F. Craparo *et al.*, "mPEG-PLGA nanoparticles labelled with loaded or conjugated Rhodamine-B for potential nose-to-brain delivery," *Pharmaceutics,* vol. 13, no. 9, p. 1508, 2021.

[45] A. Bonaccorso, T. Musumeci, M. Serapide, R. Pellitteri, I. Uchegbu, and G. Puglisi, "Nose to brain delivery in rats: Effect of surface charge of rhodamine B labeled nanocarriers on brain subregion localization," *Colloids and Surfaces B: Biointerfaces,* vol. 154, pp. 297-306, 2017.

[46] Y.-H. Zhu, J.-L. Wang, H.-B. Zhang, M. I. Khan, X.-J. Du, and J. Wang, "Incorporation of a rhodamine B conjugated polymer for nanoparticle trafficking both in vitro and in vivo," *Biomaterials science,* vol. 7, no. 5, pp. 1933-1939, 2019.

[47] J. Li, D. Liu, G. Tan, Z. Zhao, X. Yang, and W. Pan, "A comparative study on the efficiency of chitosan-N-acetylcysteine, chitosan oligosaccharides or carboxymethyl chitosan surface modified nanostructured lipid carrier for ophthalmic delivery of curcumin," *Carbohydrate polymers,* vol. 146, pp. 435-444, 2016.

[48] A. Ron, N. Davoudi, X. L. Deán-Ben, and D. Razansky, "Self-gated respiratory motion rejection for optoacoustic tomography," *Applied Sciences,* vol. 9, no. 13, p. 2737, 2019.

[49] M. Schwarz, N. Garzorz-Stark, K. Eyerich, J. Aguirre, and V. Ntziachristos, "Motion correction in optoacoustic mesoscopy," *Scientific reports,* vol. 7, no. 1, pp. 1-9, 2017.

[50] J. Aguirre *et al.*, "Motion quantification and automated correction in clinical RSOM," *IEEE transactions on medical imaging,* vol. 38, no. 6, pp. 1340-1346, 2019.

[51] Z. Cheng, H. Ma, Z. Wang, W. Zhang, F. Yang, and S. Yang, "Subpixel and on-line motion correction for photoacoustic dermoscopy," *IEEE Journal of Selected Topics in Quantum Electronics,* vol. 27, no. 4, pp. 1-8, 2020.

[52] H. Hu *et al.*, "Stretchable ultrasonic arrays for the three-dimensional mapping of the modulus of deep tissue," *Nat Biomed Eng,* May 1 2023, doi: 10.1038/s41551-023-01038-w.
