# Supplementary material for: A flexible optoacoustic blood ‘stethoscope’ for noninvasive multiparametric cardiovascular monitoring
Source: Nat Commun. 2023 Aug 4;14:4692. doi: 10.1038/s41467-023-40181-5 (PMC10403590; doi:10.1038/s41467-023-40181-5)
Supplement: Supplementary file 3 — Description of additional supplementary files [file 41467_2023_40181_MOESM3_ESM.pdf]

Description of additional supplementary files

Supplementary Movie 1: 3D imaging of blood hypoxia.

Supplementary Movie 2: 3D imaging of drug concentration decay.

Supplementary Movie 3: Perfusion experiments on human dorsal vein measured by OBS.

Supplementary Movie 4: Perfusion experiments on human dorsal vein measured by medical ultrasound imaging.

Supplementary Movie 5: Perfusion experiments on the human radial artery.

Supplementary Movie 6: Perfusion experiments on human radial artery measured by medical ultrasound imaging.

Supplementary Movie 7: FMD experiments on human radial artery using medical ultrasound imaging for cross-validation.
