# Supplementary material for: A flexible optoacoustic blood ‘stethoscope’ for noninvasive multiparametric cardiovascular monitoring
Source: Nat Commun. 2023 Aug 4;14:4692. doi: 10.1038/s41467-023-40181-5 (PMC10403590; doi:10.1038/s41467-023-40181-5)
Supplement: Supplementary file 11 — Reporting Summary [file 41467_2023_40181_MOESM11_ESM.pdf]

nature portfolio

Corresponding author(s): Zheng Yuanjin

Last updated by author(s): Jul 1, 2023

Reporting Summary

Nature Portfolio wishes to improve the reproducibility of the work that we publish. This form provides structure for consistency and transparency in reporting. For further information on Nature Portfolio policies, see our Editorial Policies and the Editorial Policy Checklist.

Statistics

For all statistical analyses, confirm that the following items are present in the figure legend, table legend, main text, or Methods section.

n/a	Confirmed	
☐	☒	The exact sample size (*n*) for each experimental group/condition, given as a discrete number and unit of measurement
☐	☒	A statement on whether measurements were taken from distinct samples or whether the same sample was measured repeatedly
☒	☐	The statistical test(s) used AND whether they are one- or two-sided<br>*Only common tests should be described solely by name; describe more complex techniques in the Methods section.*
☐	☒	A description of all covariates tested
☐	☒	A description of any assumptions or corrections, such as tests of normality and adjustment for multiple comparisons
☐	☒	A full description of the statistical parameters including central tendency (e.g. means) or other basic estimates (e.g. regression coefficient) AND variation (e.g. standard deviation) or associated estimates of uncertainty (e.g. confidence intervals)
☒	☐	For null hypothesis testing, the test statistic (e.g. *F*, *t*, *r*) with confidence intervals, effect sizes, degrees of freedom and *P* value noted<br>*Give P values as exact values whenever suitable.*
☒	☐	For Bayesian analysis, information on the choice of priors and Markov chain Monte Carlo settings
☒	☐	For hierarchical and complex designs, identification of the appropriate level for tests and full reporting of outcomes
☒	☐	Estimates of effect sizes (e.g. Cohen's *d*, Pearson's *r*), indicating how they were calculated

Our web collection on statistics for biologists contains articles on many of the points above.

Software and code

Policy information about availability of computer code

Data collection	The single-channel optoacoustic data is retrieved from the oscilloscope using a bespoke MATLAB code (version 2017b), whereas the multichannel optoacoustic imaging data is captured through a commercial Sonix DAQ. A Xilinx FPGA, programmed with Vivado version 2017.4, is utilized for synchronization purposes.
Data analysis	All optoacoustic data sets are processed using a bespoke MATLAB (2017b) program and k-wave 1.2.1.

For manuscripts utilizing custom algorithms or software that are central to the research but not yet described in published literature, software must be made available to editors and reviewers. We strongly encourage code deposition in a community repository (e.g. GitHub). See the Nature Portfolio guidelines for submitting code & software for further information.

Data

Policy information about availability of data

All manuscripts must include a data availability statement. This statement should provide the following information, where applicable:

- Accession codes, unique identifiers, or web links for publicly available datasets
- A description of any restrictions on data availability
- For clinical datasets or third party data, please ensure that the statement adheres to our policy

The main data that supporting the results of this study are available within the paper and its Supplementary Information. The data generated in this study are available from figshare: https://doi.org/10.6084/m9.figshare.23541531. Source data are provided with this paper.

Research involving human participants, their data, or biological material

Policy information about studies with human participants or human data. See also policy information about sex, gender (identity/presentation), and sexual orientation and race, ethnicity and racism.

Reporting on sex and gender	Sex and gender-based analyses were deemed irrelevant to this study, as no evidence suggests any potential influence on perfusion, venous distensibility, and flow-mediated dilation measurements.
Reporting on race, ethnicity, or other socially relevant groupings	Factors such as race, ethnicity, and other social categorizations were not considered in this study, due to a lack of evidence suggesting any potential impact on perfusion, venous distensibility, and flow-mediated dilation measurements.
Population characteristics	Healthy subjects, 1 female and 2 males, 20–30 years old.
Recruitment	Participants for the study were recruited through email invitations, ensuring a transparent and unbiased process by excluding individuals from our research group or any affiliated partnerships. Those participating in the perfusion, venous distensibility, and flow-mediated dilation studies were healthy individuals, free of any cardiovascular disease, who voluntarily consented to partake in the research.
Ethics oversight	All protocols for human trials received approval from the Nanyang Technological University Institutional Review Board (IRB), in compliance with federally approved guidelines.

Note that full information on the approval of the study protocol must also be provided in the manuscript.

Field-specific reporting

Please select the one below that is the best fit for your research. If you are not sure, read the appropriate sections before making your selection.

[x] Life sciences [ ] Behavioural & social sciences [ ] Ecological, evolutionary & environmental sciences

For a reference copy of the document with all sections, see nature.com/documents/nr-reporting-summary-flat.pdf

Life sciences study design

All studies must disclose on these points even when the disclosure is negative.

Sample size	Four nude mice were utilized for oxygen saturation and drug concentration decay tests. Three consenting participants were enlisted to validate the functionality of the device. We determined this sample size based on the typical standard in optoacoustic experiments, balancing both the need for reliable data and ethical considerations for animal usage. The small sample size successfully provides initial validation and enables us to refine the functionality of our device for further larger scale studies. Based on the results, we believe these sample sizes are sufficient for the scope and goals of this current study.
Data exclusions	No data were excluded from the study.
Replication	Experiments were repeated at least 3 times within one day for each individual and all replication attempts yielded successful results.
Randomization	In our study, subjects for the in vivo trials, which included both human participants and mice, were selected through a random process without consideration of specific attributes such as age, sex, or other potentially biasing factors. The allocation of subjects into experimental groups was purely random to minimize potential bias and control for covariates. As such, the allocation process does not introduce any systematic difference between the groups, and any observed differences in outcomes can be attributed to the variable being tested, ensuring the validity and integrity of our experimental results.
Blinding	The tasks of data collection and analysis for each subject were delegated to different researchers, ensuring an unbiased approach.

Reporting for specific materials, systems and methods

We require information from authors about some types of materials, experimental systems and methods used in many studies. Here, indicate whether each material, system or method listed is relevant to your study. If you are not sure if a list item applies to your research, read the appropriate section before selecting a response.

Materials & experimental systems

n/a	Involved in the study
☒	☐ Antibodies
☒	☐ Eukaryotic cell lines
☒	☐ Palaeontology and archaeology
☐	☒ Animals and other organisms
☒	☐ Clinical data
☒	☐ Dual use research of concern
☒	☐ Plants

Methods

n/a	Involved in the study
☒	☐ ChIP-seq
☒	☐ Flow cytometry
☒	☐ MRI-based neuroimaging

Animals and other research organisms

Policy information about studies involving animals; ARRIVE guidelines recommended for reporting animal research, and Sex and Gender in Research

Laboratory animals	Nude mice (CrTac: NCr-Foxn1nu, Male, 6-8 weeks, Invivos) were included in the study. The mice were housed under conditions of an ambient temperature between 18-26°C, relative humidity of 30-70%, and illumination of approximately 325 lux, positioned 1.0 metre above the floor for a 12-hour light/dark cycle each day.
Wild animals	This study did not involve the participation of any wild animals.
Reporting on sex	The variable of sex was not considered in this study due to its lack of influence on the results.
Field-collected samples	The study did not involve any samples collected from field research.
Ethics oversight	All animal procedures and care regimens adhered to protocols approved by the Nanyang Technological University Institutional Animal Care and Use Committee (IACUC), in accordance with federally approved guidelines.

Note that full information on the approval of the study protocol must also be provided in the manuscript.
